# Bidirectional Movement of Emerging H5N8 Avian Influenza Viruses Between Europe and Asia via Migratory Birds Since Early 2020

**DOI:** 10.1093/molbev/msad019

**Published:** 2023-01-27

**Authors:** Guogang Zhang, Bingying Li, Jayna Raghwani, Bram Vrancken, Ru Jia, Sarah C Hill, Guillaume Fournié, Yanchao Cheng, Qiqi Yang, Yuxin Wang, Zengmiao Wang, Lu Dong, Oliver G Pybus, Huaiyu Tian

**Affiliations:** Key Laboratory of Forest Protection of National Forestry and Grassland Administration, Ecology and Nature Conservation Institute, Chinese Academy of Forestry, National Bird Banding Center of China, Beijing, China; State Key Laboratory of Remote Sensing Science, Center for Global Change and Public Health, College of Global Change and Earth System Science, Beijing Normal University, Beijing, China; Department of Biology, University of Oxford, Oxford, United Kingdom; Department of Pathobiology and Population Sciences, The Royal Veterinary College, London, United Kingdom; Department of Microbiology and Immunology, Rega Institute, Laboratory of Evolutionary and Computational Virology, KU Leuven, Leuven, Belgium; Spatial Epidemiology Lab (SpELL), Université Libre de Bruxelles, Bruxelles, Belgium; Key Laboratory of Forest Protection of National Forestry and Grassland Administration, Ecology and Nature Conservation Institute, Chinese Academy of Forestry, National Bird Banding Center of China, Beijing, China; Department of Pathobiology and Population Sciences, The Royal Veterinary College, London, United Kingdom; Department of Pathobiology and Population Sciences, The Royal Veterinary College, London, United Kingdom; State Key Laboratory of Remote Sensing Science, Center for Global Change and Public Health, College of Global Change and Earth System Science, Beijing Normal University, Beijing, China; Department of Ecology and Evolutionary Biology, Princeton University, Princeton, NJ, USA; State Key Laboratory of Remote Sensing Science, Center for Global Change and Public Health, College of Global Change and Earth System Science, Beijing Normal University, Beijing, China; State Key Laboratory of Remote Sensing Science, Center for Global Change and Public Health, College of Global Change and Earth System Science, Beijing Normal University, Beijing, China; Ministry of Education Key Laboratory for Biodiversity and Ecological Engineering, College of Life Sciences, Beijing Normal University, Beijing, China; Department of Biology, University of Oxford, Oxford, United Kingdom; Department of Pathobiology and Population Sciences, The Royal Veterinary College, London, United Kingdom; State Key Laboratory of Remote Sensing Science, Center for Global Change and Public Health, College of Global Change and Earth System Science, Beijing Normal University, Beijing, China

**Keywords:** migratory birds, highly pathogenic avian influenza, intercontinental disseminations, H5N8, satellite tracking

## Abstract

Migratory birds play a critical role in the rapid spread of highly pathogenic avian influenza (HPAI) H5N8 virus clade 2.3.4.4 across Eurasia. Elucidating the timing and pattern of virus transmission is essential therefore for understanding the spatial dissemination of these viruses. In this study, we surveyed >27,000 wild birds in China, tracked the year-round migration patterns of 20 bird species across China since 2006, and generated new HPAI H5N8 virus genomic data. Using this new data set, we investigated the seasonal transmission dynamics of HPAI H5N8 viruses across Eurasia. We found that introductions of HPAI H5N8 viruses to different Eurasian regions were associated with the seasonal migration of wild birds. Moreover, we report a backflow of HPAI H5N8 virus lineages from Europe to Asia, suggesting that Europe acts as both a source and a sink in the global HPAI virus transmission network.

## Introduction

The highly pathogenic avian influenza (HPAI) H5N8 virus (H5 clade 2.3.4.4) was first detected in poultry in South Korea in 2014. By mid-2015, it had disseminated to domestic and wild birds across Asia, Europe, and North America (Hall et al. 2015).

In the first half of 2020, a new wave of HPAI H5N8 viruses caused severe poultry and wild bird outbreaks in several European countries (European Food Safety Authority et al. 2020a, 2020b; Xiong et al. 2021). Phylogenetic analyses suggested that the virus was possibly introduced to these countries from Africa by migratory birds (King et al. 2020; Swieton et al. 2020). In summer 2020, two genetically distinct HPAI H5N8 viral lineages were detected in Russia and Kazakhstan. One of these lineages was closely related to an H5N8 virus isolated in Europe in early 2020, whereas the other corresponded to a novel reassortant virus (Lewis et al. 2021; Sobolev et al. 2021). Subsequently, in the autumn and winter of 2020–2021, waves of HPAI H5N8 outbreaks swept across Europe (European Food Safety Authority et al. 2020c; Xiong et al. 2021) and East Asia (Isoda et al. 2020; Jeong et al. 2020; Li et al. 2021). In Europe, HPAI H5N8 virus outbreaks in early and late 2020 were caused by different viruses, with the later epidemic linked to the novel reassortant virus isolated in Russia and Kazakhstan (Beerens et al. 2021; Liang et al. 2021). The HPAI H5N8 virus outbreaks in East Asia (Isoda et al. 2020; Jeong et al. 2020; Baek et al. 2021; Beerens et al. 2021; Khalil et al. 2021; Xiong et al. 2021) during winter 2020–2021 shared a recent common ancestor with viruses circulating in Europe in late 2020, whereas some HPAI H5N8 viruses detected in South Korea were closely related to European viruses from early 2020 (Baek et al. 2021), indicating the seasonal spread of HPAI virus lineages across the globe (Isoda et al. 2020; Jeong et al. 2020; Sobolev et al. 2021). In December 2020, the first human case of HPAI H5N8 virus infection was reported in Russia (Pyankova et al. 2021), indicating that the virus should be closely monitored for its potential threat to public health.

In order to prepare for future HPAI outbreaks, it is essential to understand how these viruses circulate and propagate across Eurasia. As natural reservoirs of avian influenza viruses (AIVs), migratory birds gather in large numbers across broad geographic areas during migration (Altizer et al. 2011; Global Consortium for H5N8 and Related Influenza Viruses 2016), providing optimal conditions for accelerating virus dissemination. The rapid global spread of HPAI H5N8 has been linked with wild bird migration (Hall et al. 2015; Global Consortium for H5N8 and Related Influenza Viruses 2016; Fusaro et al. 2019), particularly the regular directional flight of birds between wintering and breeding sites during the northern hemisphere spring and autumn (Olsen et al. 2006). Similarly, H5N6 viruses detected in poultry in several European countries during the winter of 2017–2018 were closely associated with wild birds migrating to Europe during autumn (Beerens et al. 2019; van der Kolk 2019).

Migratory birds have been implicated in the dissemination of HPAI H5N1 in Asia (Tian et al. 2015) and from Asia to Europe (Bragstad et al. 2007; Keawcharoen et al. 2008; Breed et al. 2010; Xu et al. 2016). In addition, a study of North American birds has shown that the extent of AIV gene flow correlates with migratory flyways, with relatively frequent gene exchange occurring between regions of the same flyway (Lam et al. 2012). However, although other studies have focused on individual or partial wild bird migration flyways to evaluate their role in AIV transmission, quantitative analysis of the association between large-scale wild bird migration networks and intercontinental dissemination of AIV has been lacking (Bahl et al. 2013; Global Consortium for H5N8 and Related Influenza Viruses 2016; Fourment et al. 2017).

Multiple waves of HPAI H5N8 outbreaks in Eurasia during 2020–2021 present an opportunity to understand the spread of emerging AIVs. Our surveillance program in China provides valuable data to address key questions about HPAI H5N8 virus transmission in wild birds. Specifically, we study the seasonal pattern of HPAI H5N8 virus dissemination across Eurasia from 2020 to 2021 and investigate the role of migratory waterfowl. We combined satellite tracking data of wild birds in Eurasia (covering 20 migratory bird species in China) with virus genomic data sampled from wintering, breeding, and stopover sites in China. Our results indicate that the migratory bird network linking Europe and Asia predicts intercontinental virus spread. More importantly, we find evidence that virus lineage movement between Europe and Asia through wild bird migration is bidirectional, that is both from Asia to Europe and from Europe to Asia.

## Results

### HPAI H5N8 Seasonal Migration

We collected 27,242 samples from wild birds in China from March 2020 to May 2021, from which we obtained and sequenced a total of 65 HPAI H5N8 complete genomes, out of 74 polymerase chain reaction (PCR)-positive samples (tables 1 and 2 and supplementary table S1, Supplementary Material online). Most of the HPAI H5N8 genomes were from 2020 to 2021 wintering period (55 sequences, 52 complete genomes), whilst 11 sequences (10 complete) were from the following spring migration in 2021. Only five sequences (one complete) were sampled during the 2020 breeding and autumn migration periods, and three sequences (two complete) were sampled in the 2021 breeding period.

**Table 1.** The Number of Samples Tested from Wild Migratory Birds in Western, Central, and Eastern China from March 2020 to May 2021.

Period	Total Samples	Locations (number of samples)	Number of H5N8-Positive Samples	Number of AIV-Positive Samples
Spring migration, 2020	359	Inner Mongolia, northern China (359)	0	0
Breeding, 2020	6,699	Inner Mongolia, northern China (3,160)	1	2
		Qinghai, western China (3,539)	0	0
Autumn migration, 2020	2,789	Inner Mongolia, eastern China (1,622)	0	0
		Qinghai, Tibet and Ningxia, western China (1,167)	4	5
Wintering, 2020–2021	7,949	Shandong, eastern China (68)	0	1
		Yunnan and Tibet, western China (3,597)	1	1
		Henan and Hubei, central China (4,284)	55	56
Spring migration, 2021	6,682	Inner Mongolia, northern China (3,031)	0	0
		Tibet and Ningxia, western China (3,651)	10	10
Breeding, 2021	2,764	Tibet, western China (2764)	3	3

**Table 2.** Sampling Information on Wild Birds in Western, Central, and Eastern China from March 2020 to May 2021.

Time	Sampling Period	Site	Coordinates	Species	Type of Sample	Number of Complete HPAIV H5N8 Genomic Sequence/Number of HPAIV H5N8-Positive Samples/Number Tested	Number of Birds
March to April 2020	Spring migration	Tumuji National Nature Reserve, Inner Mongolia of northern China	122.737E, 46.070N	Geese and ducks	F	0/0/359	—
May to August 2020	Breeding	Tumuji National Nature Reserve, Inner Mongolia of northern China	122.737E, 46.070N	Geese and ducks	F	0/1/2738	—
					S	0/0/422	211
July 2020		Qinghai Lake, Qinghai of western China	99.600E, 36.533N		F	0/0/3539	—
September 2020	Autumn migration	Naqu, Tibet of western China	92.063E, 31.482N	Geese and ducks	F	0/0/359	—
					OT	0/0/5	1
		Qinghai Lake, Qinghai of western China	99.600E, 36.533N		F	0/1/253	—
September to October 2020		Tumuji National Nature Reserve, Inner Mongolia of northern China	122.737E, 46.070N		S	0/0/16	8
October 2020					F	0/0/1606	—
October 2020		Zhongning, Ningxia of western China	105.688E, 37.465N		S	1/3/550	275
December 2020	Wintering	Brahmaputra River Basin, Tibet of western China	90.633E, 29.284N	Geese and ducks	F	0/0/2425	—
					OT	0/0/8	1
				Gulls	F	0/0/11	—
October 2020 to March 2021		Sanmenxia Reservoir Area, Henan of central China	111.157E, 34.789N	Geese and ducks	OT	0/0/323	44
					F	5/5/1937	—
					S	42/44/1326	663
January 2021		Rongcheng, Shandong of eastern China	122.494E, 37.157N	Geese and ducks	OT	0/0/58	6
				Gulls		0/0/10	1
		Wang Lake, Hubei of central China	115.341E, 29.866N	Geese and ducks	F	6/6/698	—
		Dianchi Lake, Yunnan of western China	102.666E, 24.966N	Gulls	F	0/1/1,153	—
March 2021	Spring migration	Pingluo, Ningxia of western China	106.473E, 38.968N	Geese and ducks	S	5/5/288	144
		Brahmaputra River Basin, Tibet of western China	90.633E, 29.284N		F	4/5/3,363	—
April 2021		Tumuji National Nature Reserve, Inner Mongolia of northern China	122.737E, 46.070N	Geese and ducks	F	0/0/3,031	—
May 2021	Breeding	Ali, Tibet of western China	79.818E, 33.453N	Geese and ducks	F	0/0/1,011	—
		Naqu, Tibet of western China	92.063E, 31.482N		F	2/2/1,641	—
		Naqu, Tibet of western China	92.063E, 31.482N	Geese and ducks	OT	0/0/22	2
				Gulls	OT	0/1/90	9

Note.—F, fecal; OT, organ tissues (the heart, liver, spleen, lungs, kidneys, large intestine, small intestine, pancreas, cecum, trachea, stomach, and larynx of dead wild birds, but viruses were detected only in lung samples); S, swab (oropharyngeal swabs and cloacal swabs).

**Fig. 1.**
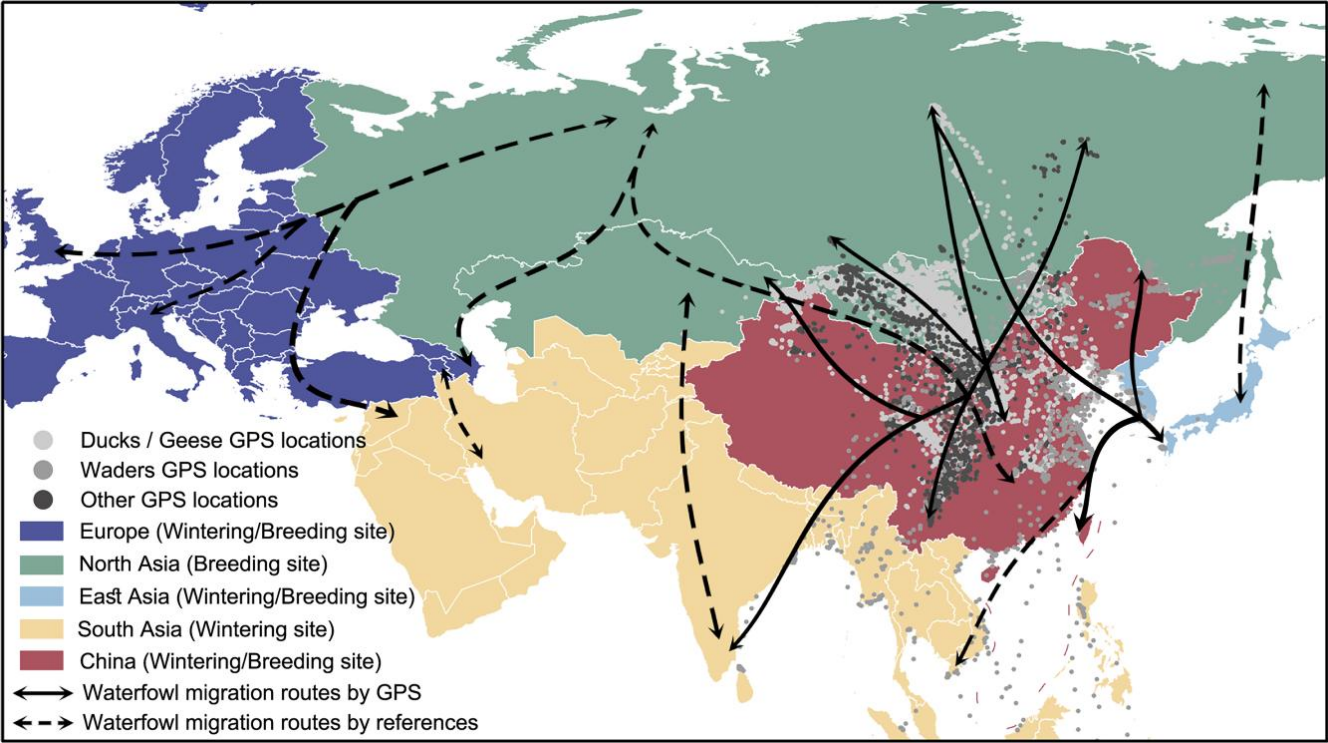
Migration flyways of wild birds in Eurasia. The dashed arrowed lines represent the migration flyways inferred from the existing literature. The solid arrowed lines represent the migration flyways observed by GPS tracking data of wild birds in China from 2006 to 2017. The gray dots show the 482,036 GPS tracking location records of 508 wild birds in China from 2006 to 2022 (an average of 948 GPS records per bird). The five geographic regions are indicated by colored shading.

Countries in Eurasia were then partitioned into five regional groups based on the type of bird residency and migration characteristics of waterfowl: Europe (wintering/breeding site), North Asia (breeding site), South Asia (wintering site), China (wintering/breeding site), and other East Asian countries (wintering/breeding site, hereafter referred to as East Asia) (fig. 1, supplementary fig. S1 and table S4, Supplementary Material online). Based on our bird trajectory global positioning system (GPS) data set and the migration routes reported in the literature, we outlined the seasonal (spring and autumn) flyways of migratory birds across Eurasia (fig. 1, supplementary tables S2 and S3, Supplementary Material online). During spring migration, many birds migrate to North Asia to breed, whereas during autumn migration, these birds migrate from North Asia to Europe, East Asia, China, and elsewhere.

To investigate the spatial spread of H5N8 viruses, we collated publicly available hemagglutinin (HA) and neuraminidase (NA) gene sequences of H5N8 viruses sampled in Europe and Asia from December 2010 to September 2021. These data sets were augmented with the 65 H5N8 complete genomes generated in this study (supplementary fig. S1, Supplementary Material online). For subsequent analyses, we curated two data sets for HA and NA using two different strategies: 1) subsampling and 2) uneven sampling (see Materials and Methods for further details). The subsampled data set was obtained by randomly selecting at most five sequences per month and region in order to examine the overall evolutionary history of HPAI H5N8 viruses. The unevenly sampled data set was collated to investigate viral diffusion among regions between 2020 and 2021 by manually selecting a few sequences per month and region in a manner that better preserved the phylogenetic temporal signal before 2020 and then randomly selecting at most ten sequences per month and region during 2020–2021.

**Fig. 2.**
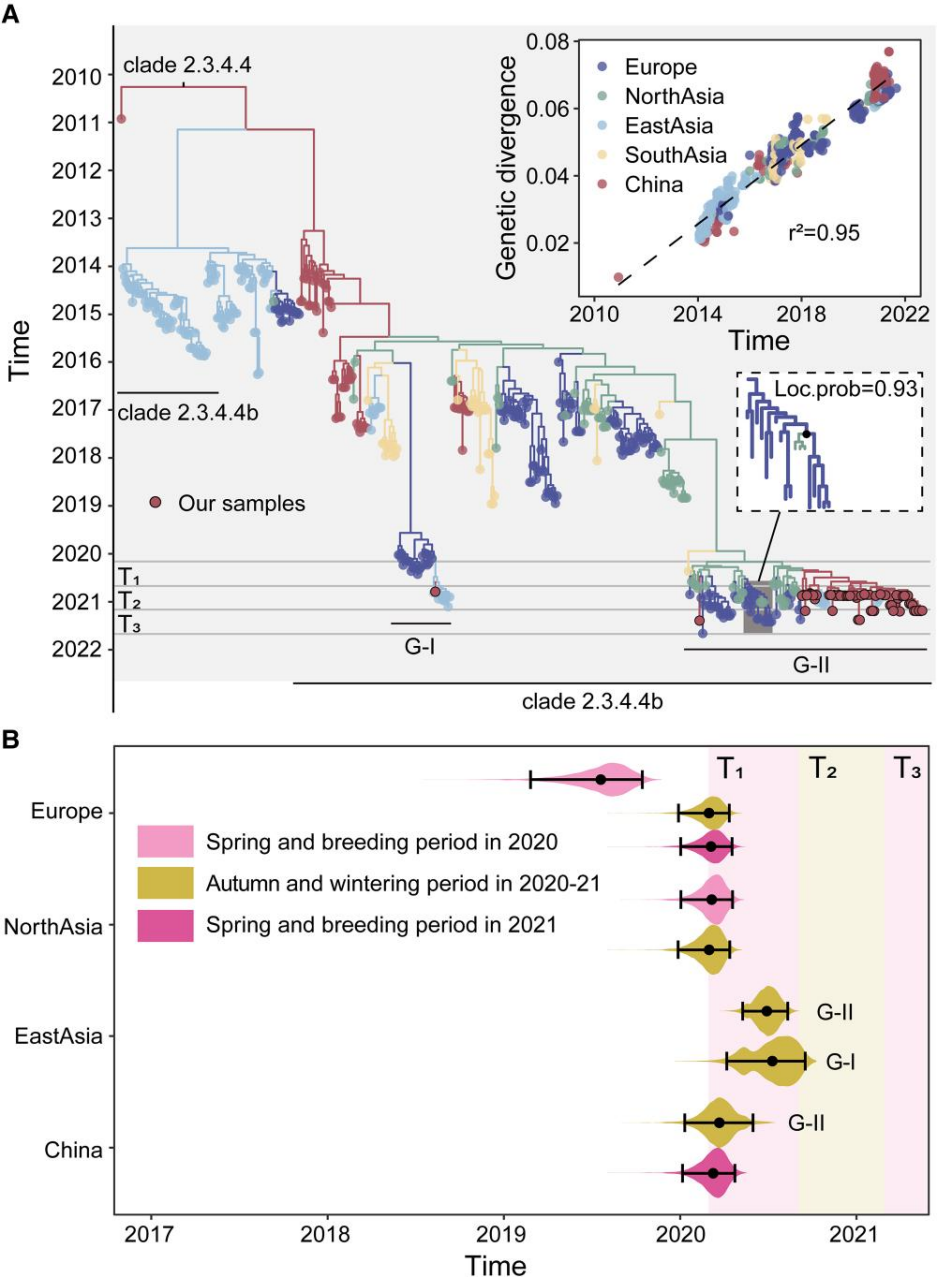
Evolution of HPAI H5N8 viruses in Eurasia. (*A*) Maximum clade credibility tree of HPAI H5N8 viruses from the subsampled HA gene data set, combined with the sequences sampled from China in this study. The colored branches indicate ancestral locations (inferred using an asymmetric discrete phylogeographic model with BSSVS). Circles drawn with a black border indicate the newly characterized genomes in this study. See the main text for the definition of time periods T1, T2, and T3, which are shown by gray vertical lines. Inset: a root-to-tip regression of genetic divergence against dates of sample collection. The geographical transition from Europe to North Asia in G-II cluster is highlighted and marked with the black dot (see bottom right inset; location posterior probability = 0.93). (*B*) The posterior distributions of tMRCAs. Violin plots represent the distribution of tMRCAs in different regions and sampling periods. The accompanying dot and the whisker plots indicate the mean and the 95% highest posterior density credible intervals for these estimates. We also used the posterior trees to infer the tMRCAs for the G-I and G-II clusters in East Asia and China. The South Asian group was excluded due to a low number of available sequences.

A time-scaled phylogeny of HA was estimated under an asymmetric discrete trait phylogeographic model (fig. 2*A*). Between January 2020 and August 2021, all detected Eurasian HPAI H5N8 viruses fell into two phylogenetic clusters within clade 2.3.4.4b (denoted G-I and G-II; fig. 2*A*). The G-I cluster was detected first in Europe in early 2020 and later in the year in East Asia and China, after the autumn migration. However, the G-I cluster was not detected in North Asia, the main migratory bird breeding site in Eurasia. This observation may reflect insufficient regional sampling. The G-II cluster was detected in South Asia and North Asia during the spring migration and breeding period in 2020, and later in Europe, China, and East Asia during and after the autumn migration period (fig. 2*A*). In addition, the G-II cluster supported a bidirectional gene flow between North Asia and Europe.

Using the HA subsampled data set, we further estimated the times of the most recent common ancestor (tMRCA) for each geographic region and each sampling period (fig. 2*B*). Sampling periods were defined by temporal phases of migration: 1) T1, the spring migration and breeding period in 2020 (March–August), 2) T2, the autumn migration and wintering period in 2020–2021 (September 2020–February 2021), and 3) T3, the spring migration and breeding period in 2021 (March–August).

The tMRCAs for viruses collected during the autumn-wintering period of 2020–2021 (T2) in East Asia occurred later than the tMRCAs for viruses collected over the same period in China, North Asia, and Europe (fig. 2*B* for HA and supplementary fig. S2, Supplementary Material online for NA). Furthermore, viruses sampled during the 2021 spring-breeding period (T3) in Europe and China exhibited similar tMRCAs, suggesting a common driver for the origin of the viruses (supplementary fig. S2, Supplementary Material online). For example, this observation could be due to the movement of migratory birds from North Asia to Europe and China during the preceding autumn migration in 2020. For Europe, tMRCAs of the 2020–2021 autumn-wintering period (T2, mean: 2020.16, 95% HPD: 2019.99–2020.28) and the 2021 spring-breeding period (T3, mean: 2020.17, 95% HPD: 2020.00–2020.29) were similar, whereas in North Asia, similar tMRCAs were observed for the 2020 spring-breeding period (T1, mean: 2020.18, 95% HPD: 2020.00–2020.30) and the 2020–2021 autumn-wintering period (T2, mean: 2020.16, 95% HPD: 2019.99–2020.28) (fig. 2*B*, supplementary fig. S2, Supplementary Material online). This observation may be consistent with the movement patterns of migratory birds: wild birds reach Europe in the autumn and outwardly migrate in the following spring to North Asia, from where they leave in the autumn (fig. 1).

### Bird Seasonal Migration

**Fig. 3.**
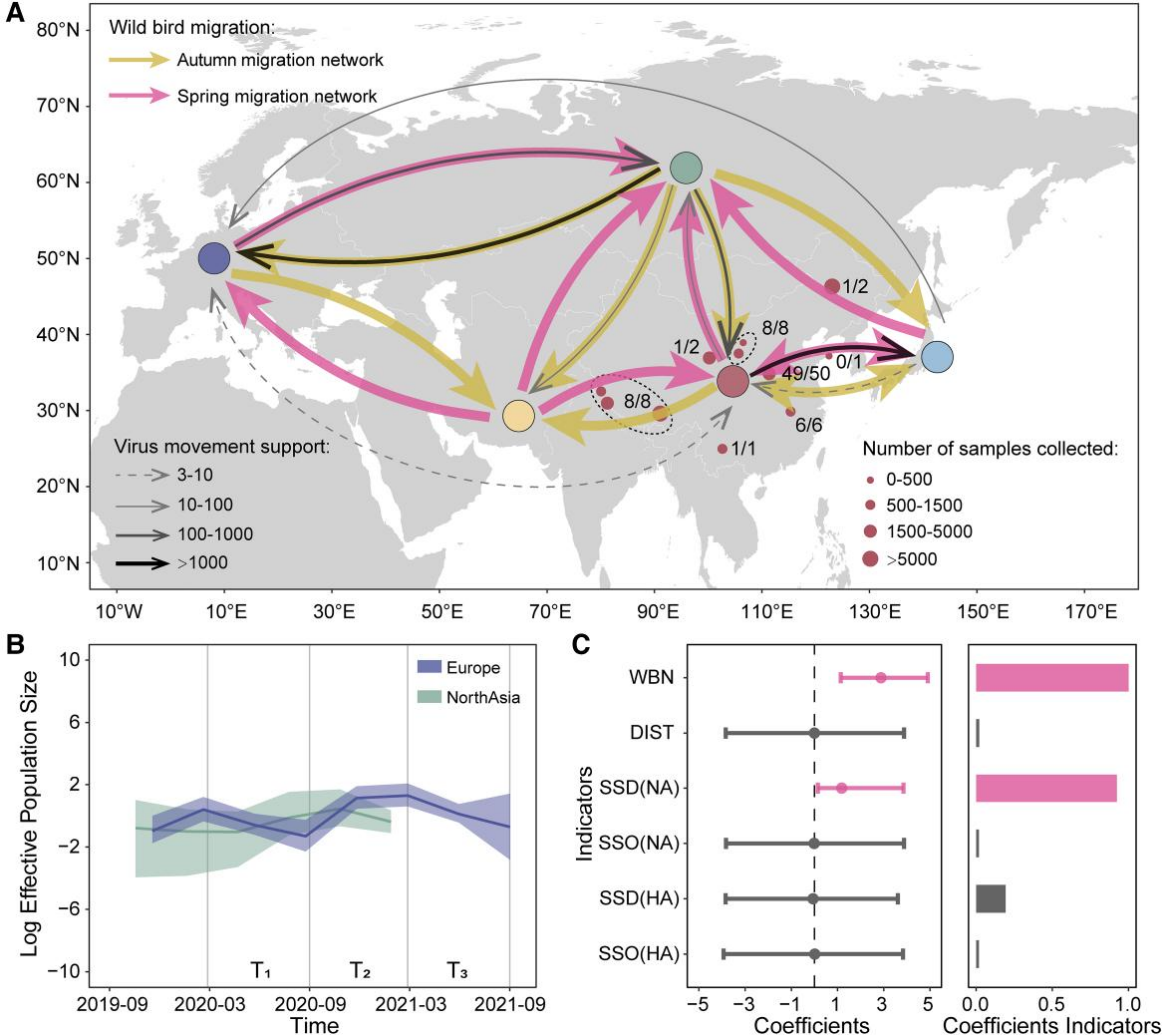
The spread of HPAI H5N8 viruses across Eurasia. (*A*) A schematic of key virus lineage movements between regions jointly inferred from the unevenly sampled HA and NA data sets (strongly supported migration rates were those with a Bayes factor ≥3 and a mean BSSVS inclusion probability ≥0.5). Colored circles indicate the five regions. The number of bird samples tested for AIV from each location in China is indicated by the size of the red circles with a black border, and the number of H5N8/AIV+ samples is indicated next to each circle. The thickness of the black arrows reflects the strength of support for virus movement (Bayes factor). The colored arrows represent the seasonal wild bird migration network in the autumn migration period (yellow) and spring migration period (pink), summarized from the GPS tracking data and existing literature. (*B*) Viral population dynamics in Europe and North Asia as estimated with the Bayesian Skygrid model using the subsampled HA and NA data sets. We define three time periods that reflect the observed trends in wild bird migration, T1, T2, and T3 (see main text and fig. 2), which are indicated by grey vertical lines. Solid lines show the mean population size estimate at each time point, and the shaded areas show the 95% highest posterior density credible intervals of that estimate. (*C*) Predictors of virus lineage movements in Eurasia jointly inferred from HA and NA using the uneven-sampled data sets. Predictors include the seasonal wild bird migration network (WBN), the great circle distance between the geographic centroids of each region (DIST); the number of virus sequences at the origin location (SSO); and the number of virus sequences at the destination location (SSD). The left-hand panel shows the coefficients of predictors (>0 for positive correlation, <0 for negative correlation), with error bars representing 95% highest posterior density credible interval. The right panel shows the posterior probability of including each predictor in the model.

Next, we undertook a joint discrete trait phylogeographic analysis of HA and NA using the unevenly sampled data sets (fig. 3*A*) to investigate the movement of viral lineage between regions in 2020–2021. Phylogeographic analysis revealed that the routes of virus lineage movement among regions were statistically significantly consistent with the migratory bird network across Eurasia (2.88, 95% HPD:1.14–4.90) (fig. 3*A*). Both the single-gene and joint HA and NA phylogeographic analyses further suggest that during 2020–2021, virus reintroduction occurred from Europe into North Asia and East Asia (figs. 2*A* and 3*A*, supplementary figs. S3–S7 and tables S5–S9, Supplementary Material online).

The mean effective population size, *N_e_*(*t*), of the HPAI H5N8 virus suggests a different demographic pattern in Europe and North Asia (fig. 3*B*), although caution is required in interpreting this result as the wide 95% HPD intervals indicate considerable uncertainty in the inferred viral population dynamics. During the spring migration and breeding period (March–August 2020, T1), *N_e_*(*t*) increased in North Asia whereas it appeared to decrease in Europe; during the 2020–2021 autumn migration and wintering period (September 2020–February 2021, T2), *N_e_*(*t*) decreased in North Asia whereas it increased in Europe. However, additional data will be necessary to validate the observed viral population dynamics in North Asia due to the large uncertainty in *N_e_*(*t*). In contrast, *N_e_*(*t*) in China and East Asia remained high over time (supplementary fig. S8, Supplementary Material online), possibly due to frequent interactions with poultry populations where the virus circulates endemically and insufficient sampling of wild birds.

We employed an epoch discrete phylogeographic model with generalized linear model (GLM) to assess the impact of the wild bird migration network on virus lineage movement. This analysis indicates that the wild bird migration network is positively associated with virus spread (2.88, 95% HPD: 1.14–4.90) and thus a key driver of HPAI H5N8 virus dispersal across Europe and Asia (fig. 3*C*). In contrast, we found no association between virus lineage movement and geographic distance between regions (fig. 3*C*). The number of virus sequences at the origin and destination sites of bird migration was incorporated as a predictor variable in the phylogeographic inference to account for sampling bias, which was found to be positively associated with virus spread for NA gene sequences (fig. 3*C*). The positive association between virus spread and the wild bird migration network was robust to the inclusion/exclusion of sample size predictors (supplementary fig. S9, Supplementary Material online). Furthermore, this result was not affected by including the spring-breeding and the autumn-wintering periods as temporal statuses in a four-epoch GLM analysis (supplementary fig. S10, Supplementary Material online).

## Discussion

Since 2020, the risk of HPAI H5N8 virus incursion through migratory waterfowl has increased in Eurasian countries (Isoda et al. 2020; King et al. 2020; Beerens et al. 2021; Sobolev et al. 2021; Xiong et al. 2021; Postel et al. 2022). By combining data on seasonal wild bird migration patterns across Eurasia with virus genomic data, our analysis suggests that the HPAI H5N8 virus has spread from Asia to Europe and back from Europe to Asia through multiple flyways.

Asia was previously thought to act as a source for HPAI H5N8 virus maintenance, from where frequent seasonal spillovers and long-distance dispersal to European sink locations occurred (Adlhoch et al. 2016; Lee et al. 2017; King et al. 2021). However, as epidemiological surveillance of wild birds has intensified and the virus epidemiology continues to shift such that infection becomes more endemic in wild birds, our understanding of HPAI H5N8 has changed. For instance, a few lineages of HPAI H5N8 viruses detected in Asia in late 2020 were genetically similar to those detected in Europe earlier that year (Isoda et al. 2020; Jeong et al. 2020; Xiong et al. 2021). Moreover, active surveillance of wild birds during 2016–2017 in the Netherlands showed that HPAI H5 clade 2.3.4.4 was still detected in European countries after migratory birds had primarily left their wintering grounds, suggesting local virus amplification in European resident birds (Poen et al. 2018). Here, we additionally confirm the backflow of HPAI H5N8 viruses from Europe to Asia. Consequently, Europe acts not only as a sink but also as a source of the global HPAI H5N8 virus transmission. This finding is similar to how our understanding of the global transmission of human influenza A viruses changes as more data accumulated. Specifically, earlier studies suggested an Asia-only source model of human influenza A (Rambaut et al. 2008) whereas later studies revealed that other regions also contribute to virus maintenance and transmission (Bedford et al. 2010; Bahl et al. 2011). In addition to this change in surveillance effort, we cannot exclude the possibility that there may have been a shift in the ecology of HPAI viruses in wild birds.

Migratory birds take multiple stops en route, and stopover sites may enable transmissions to local birds and occasionally to mammals (Briand et al. 2021; Sakuma et al. 2021; Postel et al. 2022). AIV may circulate locally and spread to other regions as wild birds migrate. North Asia is the main breeding site for wild birds in Eurasia. During spring migration, many wild birds in Europe and China migrate to North Asia for breeding, and AIV transmission may occur through contact among wild birds. Wild birds return from North Asia to Europe and China during autumn migration for wintering. We observed that the estimated tMRCAs at different periods within regions were similar to the timing of the immigration of wild birds, suggesting that the virus spreads through the seasonal migratory network. We also quantitatively assessed the impact of seasonal bird migration on the spread of the HPAI H5N8 virus across Eurasia and found that the wild bird migration network is likely to play an important role in the viral spread. Although we observed the long-distance spread of the HPAI H5N8 virus across Eurasia, this does not imply that the virus was transported the entire distance by individual birds or bird species. There is no evidence of such a long-distance migration flyway directly between China, East Asia, and Europe. The phylogenetic finding may be therefore due to insufficient sampling in the stopover and breeding sites, such as North Asia. However, some studies suggested that partial migration plays an important role in promoting the spread of the AIV between regions (Newman et al. 2009; Lee et al. 2015; Li et al. 2018; Lo et al. 2022).

Six different NA segments have been detected with HPAI H5 viruses from the 2.3.4.4 clade (de Vries et al. 2015; Hall et al. 2015). Southeast Asia and China are regions of high AIV prevalence, where multiple H5Nx viruses circulate concurrently (de Vries et al. 2015; Hall et al. 2015; Global Consortium for H5N8 and Related Influenza Viruses 2016), generating frequent opportunities for reassortment. For example, the H5N5 virus of the 2.3.4.4 clade detected in China in 2008 is likely a reassortant between the H5N1 virus and other AIVs (Gu et al. 2011; Zou et al. 2012). In 2014, a novel reassortant H5N6 virus isolated in China caused several human infections and spread to other Southeast Asian countries, possibly via migratory birds and live poultry trade (Bi et al. 2016; Thanh et al. 2018). Other reassortant viruses from the HPAI H5 2.3.4.4. clade include H5N2 and H5N3 subtypes, which have been detected in North America and Asia, respectively, and likely derived from HPAI H5N8 viruses and other AIVs.

The large effective population size of the HPAI H5N8 virus in China and East Asia is likely maintained by the frequent exchange of virus gene segments between regions via migratory birds and live poultry trade (Soares Magalhães et al. 2012; Yang et al. 2020). This may also explain why the effective population size in these regions did not change seasonally, in contrast to the effective population size in Europe and North Asia. For Southeast Asia and China, virus circulation in local poultry populations should be monitored to detect and potentially prevent genetic reassortment, which could lead to new HPAI viruses.

This study has several limitations. First, the heterogeneous sampling rates of infected wild birds across the study area and the lack of sequence data for 2019 may bias the phylogeographic inference. Additionally, the direction of movement can be obscured if the sampling of virus genomes is greater in some locations (Hill et al. 2015). Second, as we divided the year into four migration periods (wintering: November to February, spring migration: March and April, breeding: May to August, autumn migration: September and October), virus sequences were grouped into different migratory periods solely based on their sampling time. However, as migration behavior can vary among and within bird species, these groupings are likely oversimplified. Furthermore, our migratory network is not specific to any migratory bird species, as detailed seasonal migration tracking data for bird species were lacking. Thirdly, although the temporal variations in effective population size may not be statistically significant given their wide credible intervals, it is challenging to further refine these estimates with limited observed viral genetic diversity (Hall et al. 2016). Finally, the fresh fecal and swabs samples were centrifuged in sterile phosphate-buffered saline (PBS) alone, which may reduce the sensitivity of virus detection compared with the PBS with glycerol (optionally with antibiotics) as WHO guidelines recommend (Zhang et al. 2015). However, the isolation rate for our swab samples was 4.00% (52/1,301) (table 1), which is not a low isolation rate compared with other studies (Chen et al. 2006, 2019; Baumer et al. 2010; Sivay et al. 2012; Lewis et al. 2013; Na et al. 2021).

Our results confirmed that introductions of HPAI H5N8 virus lineages are positively associated with the seasonal migratory network. Moreover, we report a bidirectional gene flow of the HPAI H5N8 virus between Europe and Asia. We suggest that future efforts should aim to strengthen the epidemiological monitoring of migratory birds and clarify the susceptibility of wild bird species to different AIV strains. Regions with high AIV prevalence should also carry out active virologic surveillance of wild birds and local poultry to monitor the spread of the HPAI H5N8 virus.

## Materials and Methods

### New AIV Genomes from Wild Birds in China

In order to investigate the seasonal pattern of HPAI H5N8 virus transmission, we collected and tested a total of 27,242 samples from wild migratory birds. Seasonal bird migration consists of four periods: spring migration (March and April), breeding (May to August), autumn migration (September and October), and wintering (November to February) periods. We collected samples from feces, oropharyngeal swabs, cloacal swabs of wild birds, and lungs (heart, liver, spleen, kidneys, large intestine, small intestine, pancreas, cecum, trachea, stomach, and larynx were also collected, but positive viruses were only detected in lung samples) of dead wild birds during six periods from March 2020 to May 2021 (tables 1 and 2).

We collected 24,124 fresh fecal and 2,602 swab samples and individually dipped them in sterile PBS. The solid tissue samples (516 samples) were stored individually in plastic Ziploc bags. All samples are transported to the laboratory at cryogenic temperatures (−20 to −10 °C) and then stored at a low temperature (−80 °C) until further use.

The fresh fecal and swabs samples were centrifuged in sterile PBS, and supernatants were taken. Virus RNA was extracted from supernatants using the T014 Virus DNA/RNA Extraction Kit (Xi’an Tianlong Technology, China) and GeneRotex96 Nucleic Acid Extraction System (Xi’an Tianlong Technology, China) following the manufacturer's protocol. This magnetic bead-based kit can efficiently extract nucleic acid from cotton swab samples, and the results are consistent and repeatable even for complex low-copy samples (Yoon et al. 2016). We used the Animal Tissue Genomic DNA Extraction Kit to extract the viral RNA from tissue samples.

The extracted viral RNA was tested for AIV RNA presence using real-time quantitative PCR (only the samples with the *C*_t_ value ≤25 were selected for sequencing). The RNA was reverse transcribed with the primers and probe to conserved influenza A virus sequences, as implemented by the commercial Influenza A virus Detection Kit (Xi’an Tianlong Technology, China) in a 50-μl reaction volume. The PCR conditions comprised an initial denaturation at 42 °C insulation for 60 min, 94 °C initial denaturation for 2 min; 5 cycles of 94 °C for 30 s, 44 °C for 30 s, and 68 °C for 3 min; 40 cycles of 94 °C for 30 s, 57 °C for 30 s, and 68 °C for 3 min; and a final extension at 72 °C for 10 min. Five microliters of the product was used for electrophoresis (U: 200 V; A; 200A; T: 70 min) to confirm the presence of the target fragment. DNA concentration of each PCR amplicon was quantified using a Qubit3.0 fluorometer. The PCR products with concentrations >1 ng/μl were diluted to 0.2 ng/μl, and those with concentrations <0.2 ng/μl were discarded.

Sequencing was conducted using the Sequencing-By-Synthesis of the Illumina MiSeq system (Illumina Inc., Shanghai, China). The dsDNA was processed by cutting, and the adaptor connects using the transposome, modifying the adapter by low-cycle amplification. The sequencing primer binding site1/2, INDEX1/INDEX2 (primer [N7XX] and primer [S5XX]) oligonucleotide sequences of P5 and P7 were added. After product purification, the DNA fragments were used to construct a DNA library for high-throughput sequencing for each sample. The DNA libraries were sequenced on an Illumina MiSeq Sequencer. We performed de novo assembly and combined the results with a reference-based assembly to obtain the best assembly. All virus sequences were subtyped by searching against the NCBI nonredundant database. In total, 78 samples were positive for AIV, and 65/74 (65 complete) HPAI H5N8 virus genome sequences were obtained (supplementary fig. S1, Supplementary Material online).

### Wild Bird Migration Routes

We used a data set of satellite-tracked bird movements to get detailed migratory pathways of birds within and through China. Only migratory birds with more than 50 GPS records will be included in the data set. From 2006 to 2022, 508 wild birds were captured in 13 sites, including 12 sites in central, western, and eastern China and Ulz River in eastern Mongolia. Among the 508 wild birds captured were 3 cranes, 10 storks, 385 birds from 12 different geese and duck species, 88 birds from 2 gull species, and 22 birds from 4 shorebird species (supplementary table S3, Supplementary Material online). Each bird was tagged with a GPS transmitter using a backpack method to obtain detailed information about the bird migration route. Different satellite logger devices were used for migratory bird species with different body masses (YH-GTG0306, YH-GTG0312, YH-GTG0317, YH-GTG0325, and YH-GTG0330) (Hangzhou Yuehai Technology of China, Zhejiang). The devices were solar-powered and programmed to record the position of birds by GPS every 2 h and continuously document location data acquisition via the global system for mobile communications. As the H5N8 viruses have been reported in many Eurasian countries, we also refer to the relevant wild bird migration routes in existing literature (supplementary table S2, Supplementary Material online).

### Phylogenetic Analysis

The HA and NA gene segments of H5N8 viruses from all hosts with collection date and location information were downloaded from Global Initiative on Sharing All Influenza Data (GISAID) as of October 2021. These sequences were combined with the viral gene sequences generated here (supplementary table S1, Supplementary Material online).

We aligned the sequences using MAFFT (Katoh and Standley 2013) and reassortant sequences were detected using RDP4 (Martin et al. 2015). We identified and removed sequences with inconsistent levels of genetic divergence given their sampling time from our data sets by estimating a maximum likelihood tree using IQ-Tree (Nguyen et al. 2015) under the automatically determined best-fit substitution model and performing a root-to-tip regression analysis in TempEst v1.5.3 (Rambaut et al. 2016). According to sampling location, we divided the data sets into five groups based on the residential types and migration characteristics of waterfowl: Europe (wintering/breeding site), North Asia (breeding site), South Asia (wintering site), China (wintering/breeding site), and other East Asian countries (wintering/breeding site, further referred to as East Asia) (fig. 1, supplementary tables S2 and S4, Supplementary Material online). The data sets were downsampled using two strategies. 1) subsampling: each gene segment data set was randomly selecting at most five sequences per month and region during 2010–2021 to examine the overall evolutionary history of HPAI H5N8 viruses (“subsampled data set”) and 2) uneven sampling: in order to investigate viral diffusion among regions between 2020 and 2021, we manually selected a few sequences with strong temporal signals to better preserve the temporal signal of the data set before 2020 and then randomly selecting at most ten sequences per month and region during 2020–2021 (“uneven-sampled data set”). The final H5N8 data sets were 1) 501 HA gene sequences for the subsampled data set from December 2010 to August 2021, 2) 249 HA gene sequences for the uneven-sampled data set from April 2010 to August 2021, 3) 484 NA gene sequences for the subsampled data set from April 2010 to September 2021, and 4) 279 NA gene sequences for the uneven-sampled data set from April 2010 to August 2021 (supplementary fig. S1, Supplementary Material online).

The phylogeographic diffusion patterns were inferred with the Bayesian phylogenetic package BEAST v1.10.4 (Suchard et al. 2018) with the BEAGLE (Ayres et al. 2012) library to improve computational speeds. The analyses were performed under the SRD06 substitution model (Global Consortium for H5N8 and Related Influenza Viruses 2016), using an uncorrelated lognormal relaxed clock (Global Consortium for H5N8 and Related Influenza Viruses 2016) and the Gaussian Markov random field Bayesian Skygrid coalescent model (Hill et al. 2015) with the Bayesian Stochastic Search Variable Selection (BSSVS) algorithm. An asymmetric substitution model was used for the location discrete trait, which allows different rates of lineage movement between each pair of locations (Lemey et al. 2009). Three independent Markov Chain Monte Carlo (MCMC) runs were performed for 400 million steps and logged every 20,000 steps. The first 10% of the chains were discarded as burn-in. We confirmed the convergence of all chains in Tracer v1.7.1 (Rambaut et al. 2018), ensuring the Effective Sample Size (ESS) was >200 for all parameters. Maximum clade credibility trees were estimated using TreeAnnotator v1.10.4 and visualized using FigTree v1.4.4 (http://tree.bio.ed.ac.uk/software/figtree).

To quantify the contribution of wild bird migration routes to virus dispersal between different locations in Europe and Asia and to maximize the statistical power of our data sets, we undertook a joint analysis of HA and NA for both past population reconstruction and phylogeographic analysis. For demographic reconstruction analysis, we used a separate SRD06 substitution model (Global Consortium for H5N8 and Related Influenza Viruses 2016) and shared a Bayesian Skygrid coalescent model (Hill and Baele 2019) for HA and NA data sets. For phylogeographic analysis, we used a separate exponential coalescent prior (Griffiths and Tavare 1994; Drummond et al. 2002) and the same phylogeographic substitution models for HA and NA data sets. The GLM extension of Bayesian phylogeographic inference was applied to the uneven-sampled gene data set of HA and NA under time-heterogeneous (epoch) models (Bielejec et al. 2014). We divided each year into four periods: wintering (November to February), spring migration (March and April), breeding (May to August), and autumn migration (September and October). The predictors include 1) seasonal wild bird migration networks (we specify a value of 1 between regions with wild bird migration routes and 0 for those without; we used three migration networks, i.e., the spring migration network, the fall migration network, and a static network for the breeding and overwintering periods), 2) sample size of gene sequences at the origin and destination sites, and 3) great circle distance between the geographic centroids of each region. For each data set, three independent MCMC runs were executed for 400 million steps with a burn-in of 10%, and samples were logged every 20,000 steps. Finally, we ascertained the convergence performance of the chains in Tracer v1.7.1 (Rambaut et al. 2018).

## Supplementary Material

msad019_Supplementary_Data
