# Supplementary material for: Bidirectional Movement of Emerging H5N8 Avian Influenza Viruses Between Europe and Asia via Migratory Birds Since Early 2020
Source: Mol Biol Evol. 2023 Jan 27;40(2):msad019. doi: 10.1093/molbev/msad019 (PMC9922686; doi:10.1093/molbev/msad019)
Supplement: msad019_Supplementary_Data [file msad019_supplementary_data.docx]

Supplementary Material

Figs. S1 to S10

Tables S1 to S9


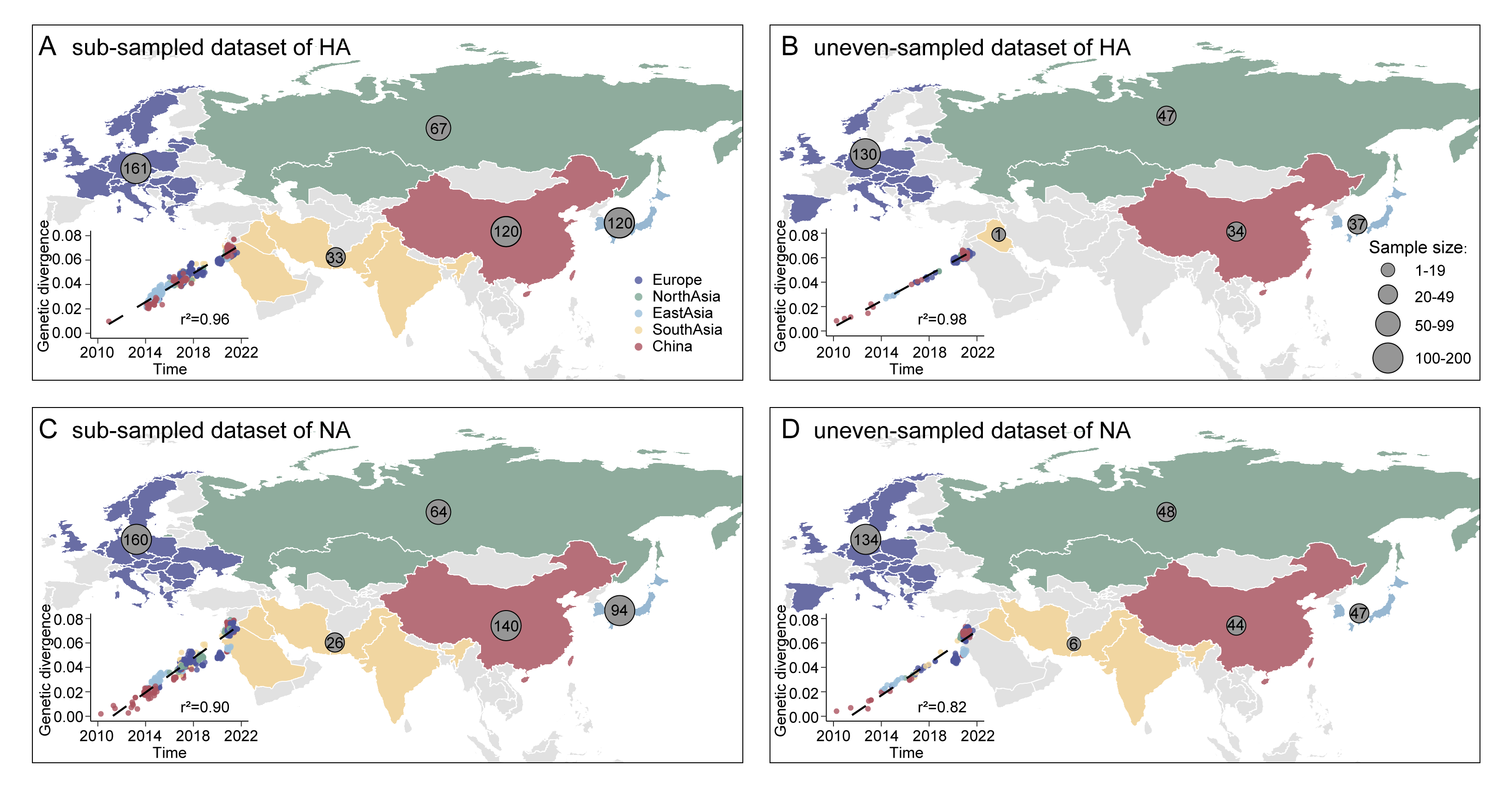


Fig. S1 The spatial distribution of the avian influenza H5N8 virus gene sequences datasets. (A) A total number of 501 HA gene sequences of the H5N8 virus for the sub-sampled dataset from December 2010 to August 2021(China:120, East Asia: 120, Europe: 161, North Asia: 67, South Asia: 33). (B) A total number of 249 HA gene sequences of the H5N8 virus for the uneven-sampled dataset from April 2010 to August 2021 (China:34, East Asia: 37, Europe: 130, North Asia: 47, South Asia: 1). (C) A total number of 484 NA gene sequences of the H5N8 virus for the sub-sampled dataset from April 2010-September 2021 (China:140, East Asia: 94, Europe: 160, North Asia: 64, South Asia: 26). (D) A total number of 279 NA gene sequences of the H5N8 virus for the uneven-sampled dataset from April 2010-August 2021 (China:44, East Asia: 47, Europe: 134, North Asia: 48, South Asia: 6). These countries were divided into five groups: Europe (purple), North Asia (green), South Asia (yellow), East Asia (blue), and China (red). Inset: a root-to-tip regression of genetic divergence against dates of sample collection.


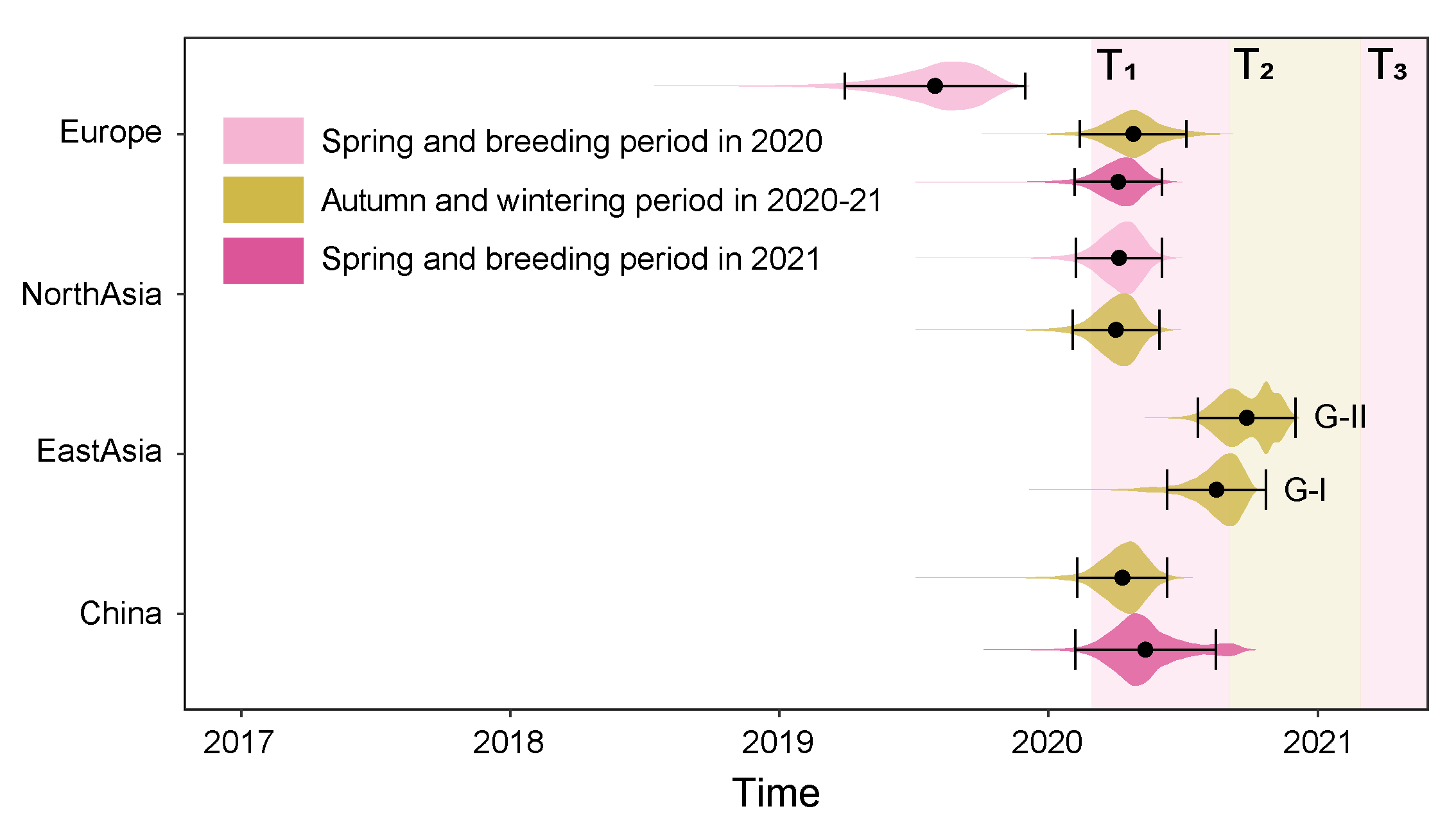


Fig. S2 The posterior distributions of time to the most recent common ancestor (tMRCA) of viral introductions for different regional groups estimated with the sub-sampled NA gene segment dataset. Violins represent tMRCAs for different regional groups during three time periods (T_1_, T_2_, T_3_ – shown in pink and goldenrod shade): spring migration and breeding period in 2020 (T_1_, pale pink), autumn migration and wintering period in 2020-21 (T_2_, golden brown), spring migration and breeding period in 2021 (T_3_, dark pink). The black dots and the whiskers in violins represent the mean values and the 95% highest posterior density credible interval for these estimates. We computed tMRCAs on the two genetic clusters separately. South Asian group was excluded due to the low number of available viral sequences.


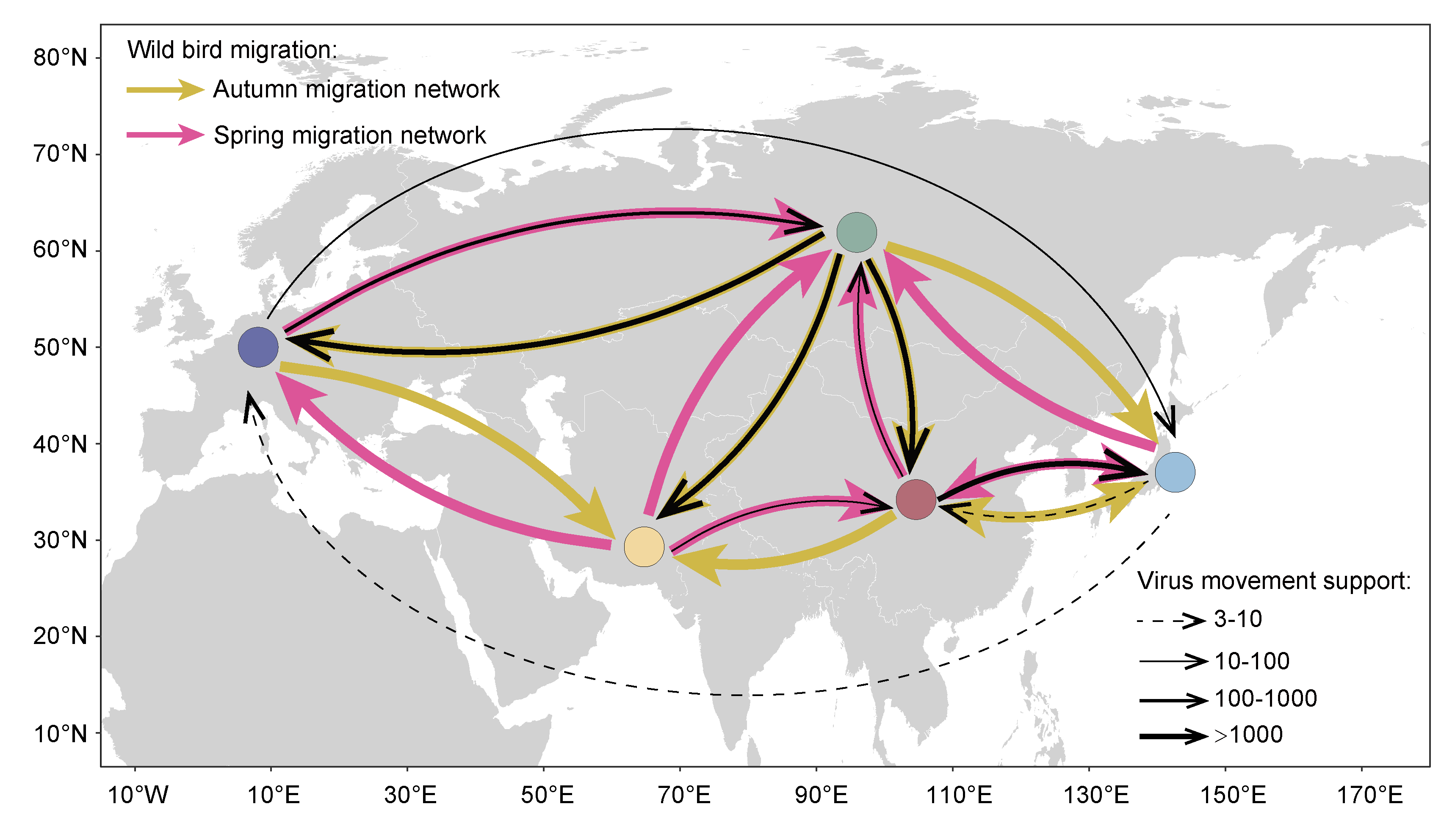


Fig. S3 A schematic of key virus lineage movements between regions, inferred from phylogeographic analysis of the sub-sampled HA gene dataset (significant migration rates were those with the Bayes factor value of ≥3 and the mean value of BSSVS inclusion probability ≥0.5). Coloured circles indicate the five regions (purple: Europe; green: North Asia; yellow: South Asia; blue: East Asia; red: China). The thickness of the black arrows reflects the strength of the virus movement support (Bayes factor). The coloured arrows represent the seasonal wild bird migration network in the autumn migration period (yellow) and spring migration period (pink), summarized from the GPS tracking data and existing literature.


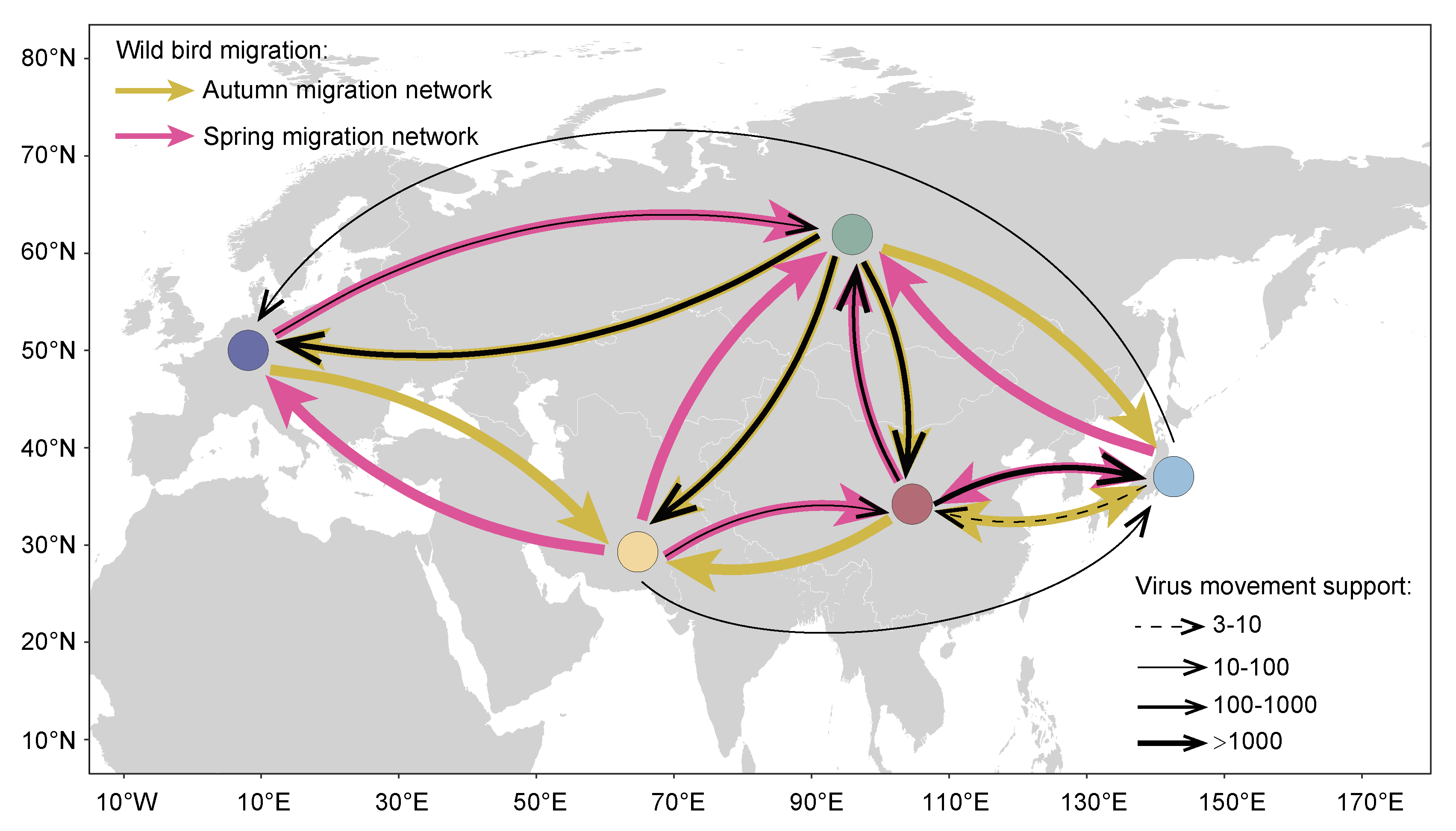


Fig. S4 A schematic of key virus lineage movements between regions, inferred from phylogeographic analysis of the sub-sampled NA gene dataset (significant migration rates were those with the Bayes factor value of ≥3 and the mean value of BSSVS inclusion probability ≥0.5). Coloured circles indicate the five regions (purple: Europe; green: North Asia; yellow: South Asia; blue: East Asia; red: China). The thickness of the black arrows reflects the strength of the virus movement support (Bayes factor). The coloured arrows represent the seasonal wild bird migration network in the autumn migration period (yellow) and spring migration period (pink), summarized from the GPS tracking data and existing literature.


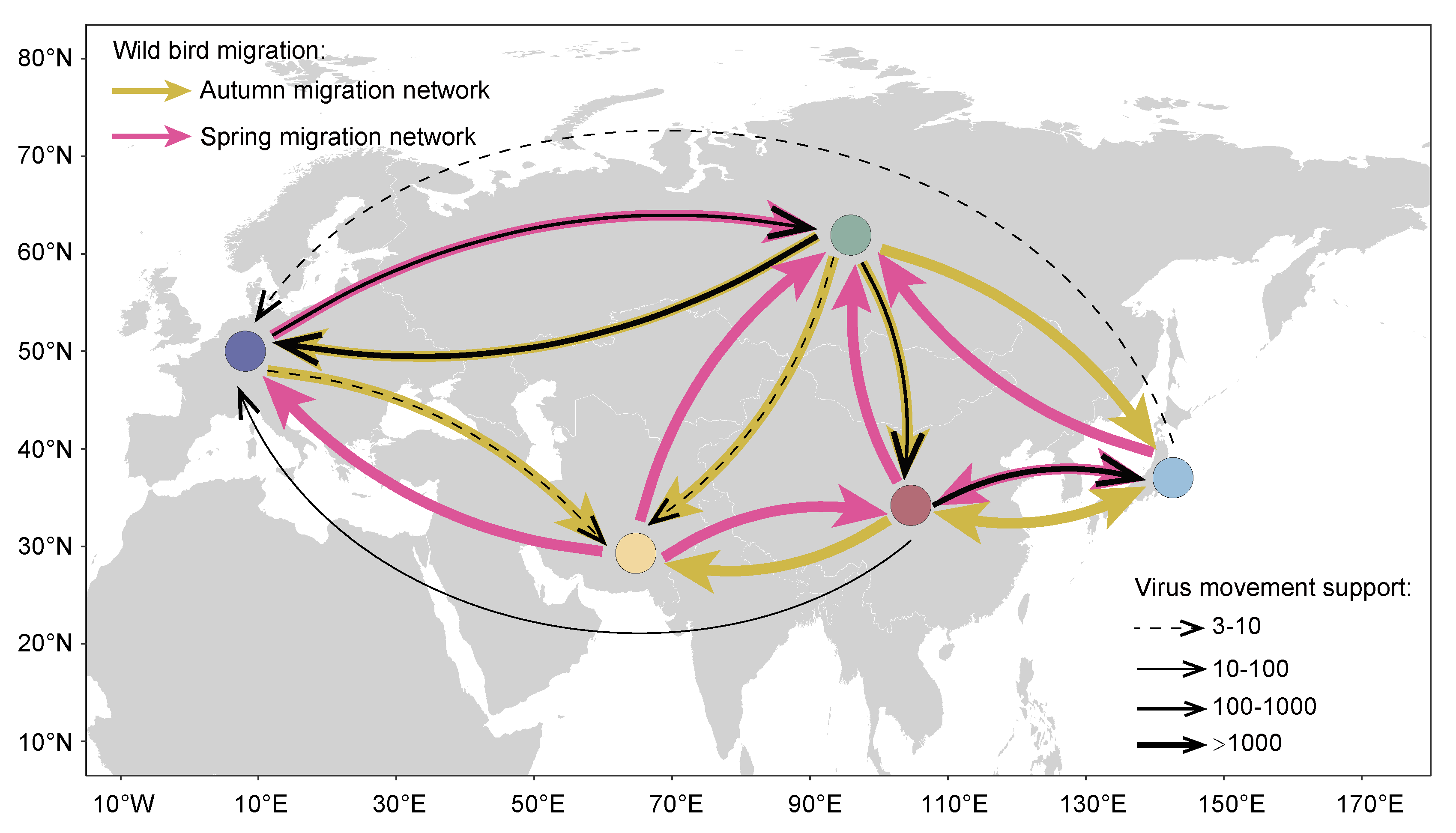


Fig. S5 A schematic of key virus lineage movements between regions, inferred from phylogeographic analysis of the uneven-sampled HA gene dataset (significant migration rates were those with the Bayes factor value of ≥3 and the mean value of BSSVS inclusion probability ≥0.5). Coloured circles indicate the five regions (purple: Europe; green: North Asia; yellow: South Asia; blue: East Asia; red: China). The thickness of the black arrows reflects the strength of the virus movement support (Bayes factor). The coloured arrows represent the seasonal wild bird migration network in the autumn migration period (yellow) and spring migration period (pink), summarized from the GPS tracking data and existing literature.


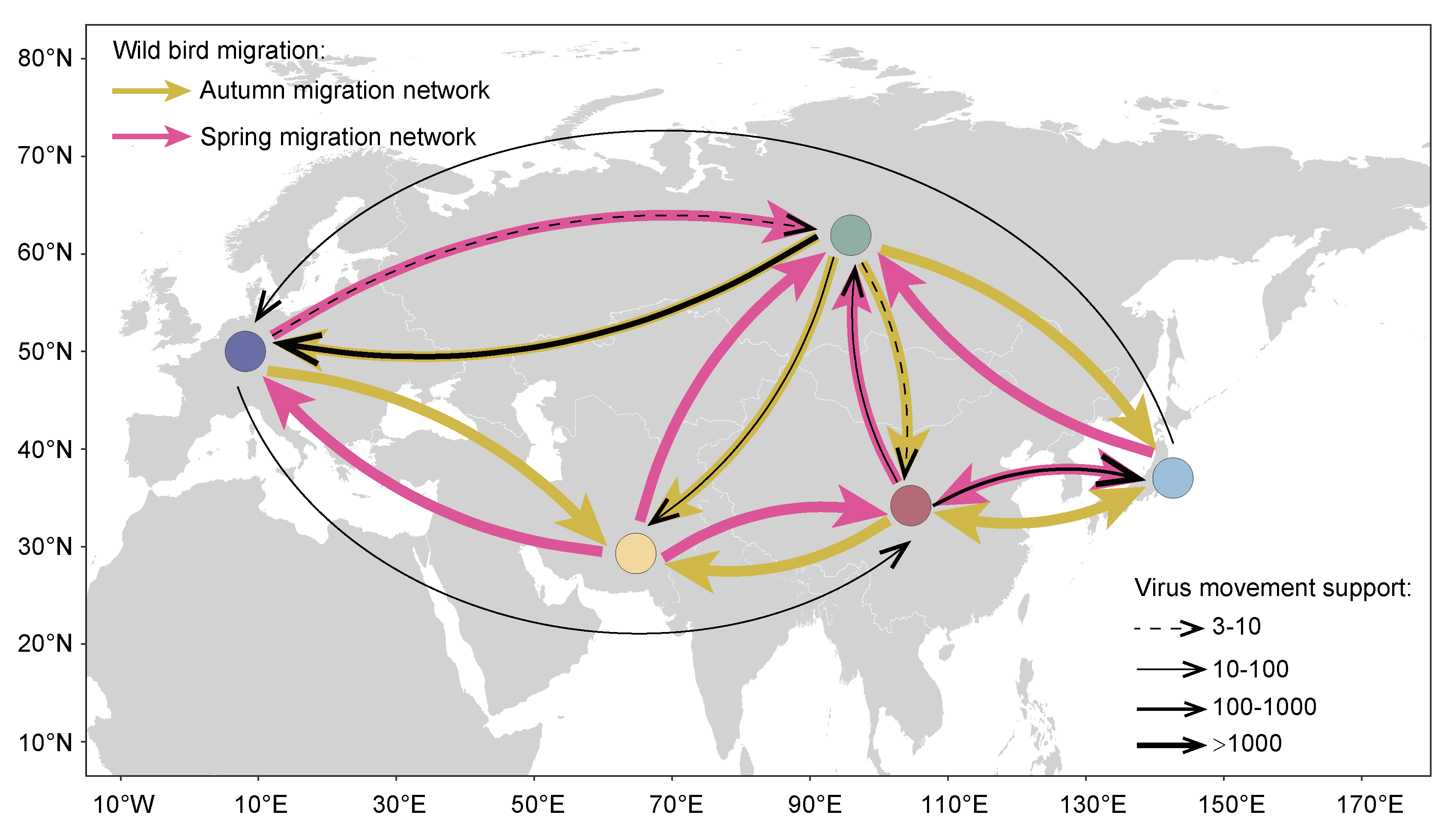


Fig. S6 A schematic of key virus lineage movements between regions, inferred from phylogeographic analysis of the uneven-sampled NA gene dataset (significant migration rates were those with the Bayes factor value of ≥3 and the mean value of BSSVS inclusion probability ≥0.5). Coloured circles indicate the five regions (purple: Europe; green: North Asia; yellow: South Asia; blue: East Asia; red: China). The thickness of the black arrows reflects the strength of the virus movement support (Bayes factor). The coloured arrows represent the seasonal wild bird migration network in the autumn migration period (yellow) and spring migration period (pink), summarized from the GPS tracking data and existing literature.


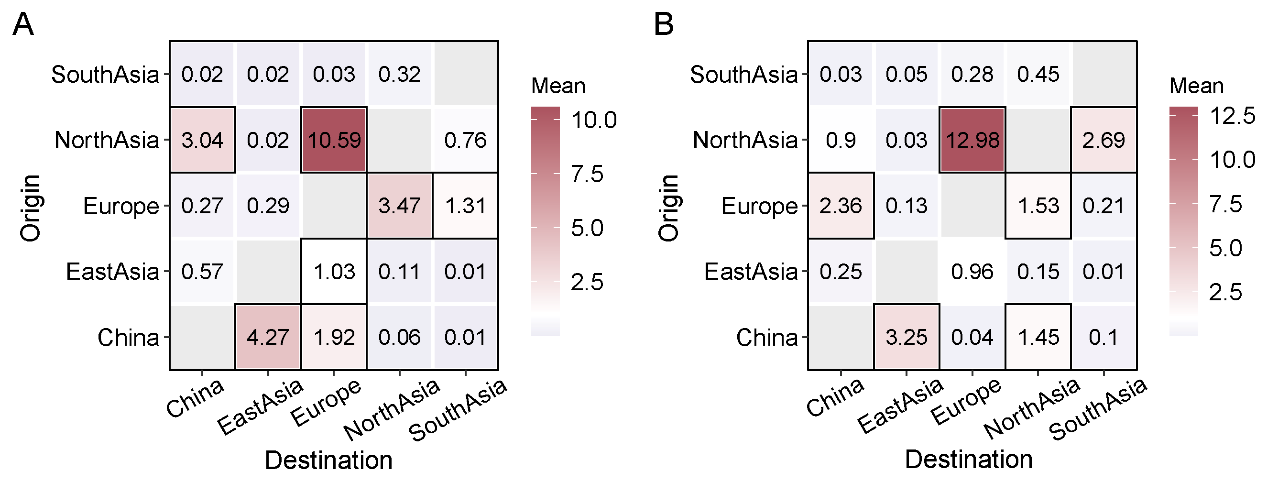


Fig. S7 Markov jump counts matrix of HPAI H5N8 virus between regions. (A) The mean Markov jump counts of HPAI H5N8 virus between regions using uneven-sampled HA gene dataset. (B) The mean Markov jump counts of HPAI H5N8 virus between regions using uneven-sampled NA gene dataset. The y-axis represents the original regions and the x-axis represents the destinations. Markov jump counts over 1 are marked on the grid with a black border respectively.


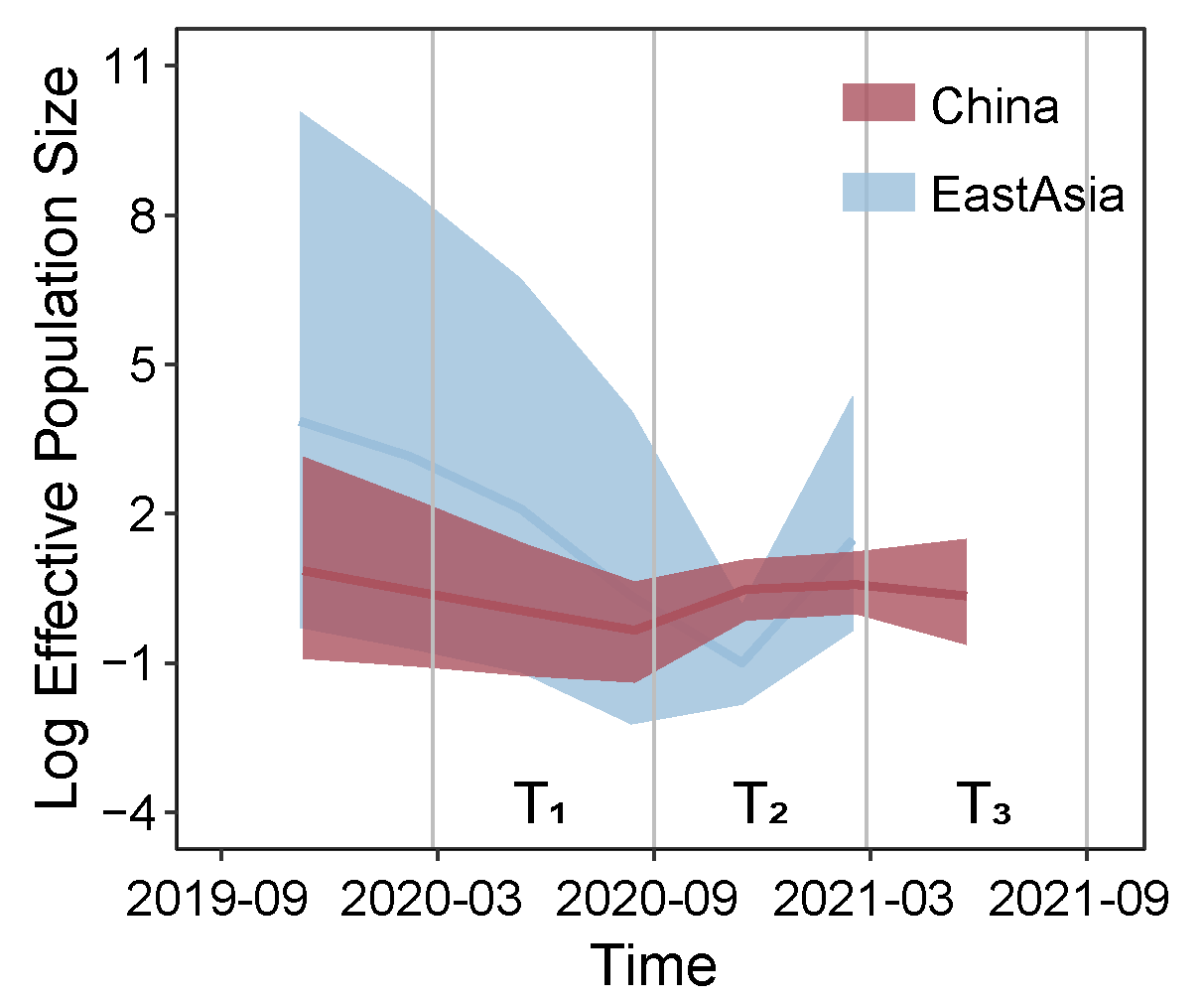


Fig. S8 Viral population dynamics in China and East Asia as estimated with the Bayesian Skygrid method by joint analysis of the sub-sampled HA and NA gene datasets. We define three time periods that reflect the observed trends in wild bird migration, T1, T2, and T3 (see main text), which are indicated by gray vertical lines. Solid lines show the mean population size estimate at each time point, and the shaded areas show the 95% highest posterior density credible intervals of that estimate.


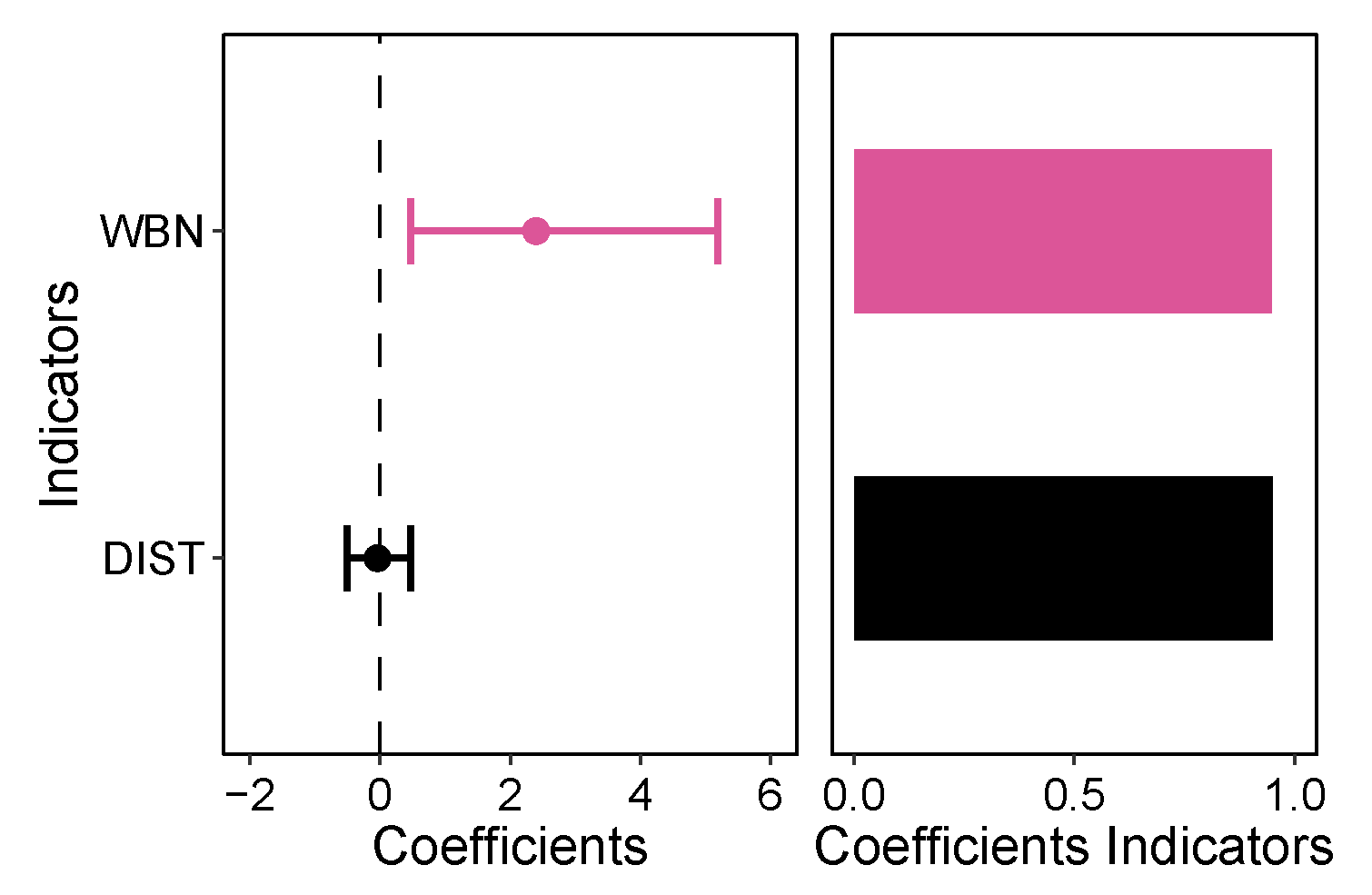


Fig. S9 Predictors of virus lineage movement in Eurasia. Predictors include the seasonal wild bird migration network (WBN), the great circle distance between the geographic centroids of each region (DIST). The left-hand panel shows the coefficients of predictors (>0 for positive correlation, <0 for negative correlation), with error bars representing 95% highest posterior density credible interval. The right panel shows the posterior probability of including each predictor in the model.


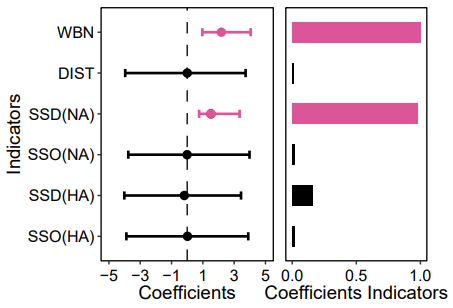


Fig. S10 The four-epoch discrete trait diffusion model extended with GLM of the viral spread between regions, inferred from joint phylogeographic analysis of the uneven sampled HA and NA gene datasets. We regard the period before the spring migration in 2020 as the first epoch, the spring-breeding period in 2020 as the second epoch, the autumn-wintering period in 2020-21 as the third epoch, and the spring-breeding period in 2021 as the fourth epoch. Predictors include the seasonal wild bird migration network (WBN), the great circle distance between the geographic centroids of each region (DIST); the number of virus sequences at the origin location (SSO); the number of virus sequences at the destination location (SSD). The left-hand panel shows the coefficients of predictors (>0 for positive correlation, <0 for negative correlation), with error bars representing 95% highest posterior density credible interval. The right panel shows the posterior probability of including each predictor in the model.

Table S1. The avian influenza viruses isolated from western, central, and eastern China along wild bird migration routes from May 2020 to May 2021.

| Position | Province | Isolate Name | Isolate ID | Collection date | Migration Period | Type of sample* | NS | PB2 | PB1 | PA | HA | NP | NA | M |
| --- | --- | --- | --- | --- | --- | --- | --- | --- | --- | --- | --- | --- | --- | --- |
| Northern China | Inner Mongolia | A/wild bird/Tumuji/TJ1533/2020(H5N8) | EPI_ISL_14819793 | 2020-05-25 | Breeding period in 2020 (May to August) | F | 1^⁑^ | 1 | - | - | 1 | 1 | 1 | 1 |
| Western China | Qinghai | A/Bar-headed Goose/Qinghai/Q24/2020(H5N8) | EPI_ISL_14819814 | 2020-09-24 | Autumn migration period in 2020 (September and October) | F | 1 | 1 | 1 | 1 | - | - | 1 | 1 |
|  | Yunnan | A/Black-headed Gull/Yunnan/D197/2021(H5N8) | EPI_ISL_14819894 | 2021-01-27 | Wintering period in 2020-2021(November in 2020 to February in 2021) | F | 1 | 1 | 1 | 1 | 1 | - | 1 | 1 |
|  | Ningxia | A/Mallard/Ningxia/BY246/2020(H5N8) | EPI_ISL_14819895 | 2020-10-16 | Autumn migration period in 2020 (September and October) | O | 1 | 1 | 1 | 1 | 1 | 1 | 1 | 1 |
|  |  | A/Mallard/Ningxia/BY247/2020(H5N8) | EPI_ISL_14819896 | 2020-10-16 |  | O | 1 | - | 1 | - | 1 | 1 | 1 | - |
|  |  | A/Mallard/Ningxia/BY249/2020(H5N8) | EPI_ISL_14819897 | 2020-10-16 |  | O | 1 | - | - | - | 1 | 1 | 1 | 1 |
|  |  | A/Wild Duck/Ningxia/Y95/2021(H5N8) | EPI_ISL_14819898 | 2021-03-09 | Spring migration period in 2021(March and April) | O | 1 | 1 | 1 | 1 | 1 | 1 | 1 | 1 |
|  |  | A/Wild Duck/Ningxia/Y99/2021(H5N8) | EPI_ISL_14819899 | 2021-03-09 |  | O | 1 | 1 | 1 | 1 | 1 | 1 | 1 | 1 |
|  |  | A/Spot-billed Duck/Ningxia/Y16/2021(H5N8) | EPI_ISL_14819900 | 2021-03-11 |  | O | 1 | 1 | 1 | 1 | 1 | 1 | 1 | 1 |
|  |  | A/Spot-billed Duck/Ningxia/Y26/2021(H5N8) | EPI_ISL_14822356 | 2021-03-11 |  | O | 1 | 1 | 1 | 1 | 1 | 1 | 1 | 1 |
|  |  | A/Wild Duck/Ningxia/Y54/2021(H5N8) | EPI_ISL_14822357 | 2021-03-11 |  | O | 1 | 1 | 1 | 1 | 1 | 1 | 1 | 1 |
|  | Tibet | A/Bar-headed Goose/Tibet/P2098/2021(H5N8) | EPI_ISL_14822358 | 2021-03-11 |  | F | 1 | 1 | 1 | 1 | 1 | - | 1 | 1 |
|  |  | A/Bar-headed Goose/Tibet/P2320/2021(H5N8) | EPI_ISL_14822426 | 2021-03-11 |  | F | 1 | 1 | 1 | 1 | 1 | 1 | 1 | 1 |
|  |  | A/Bar-headed Goose/Tibet/P1908/2021(H5N8) | EPI_ISL_14822451 | 2021-03-12 |  | F | 1 | 1 | 1 | 1 | 1 | 1 | 1 | 1 |
|  |  | A/Bar-headed Goose/Tibet/P1910/2021(H5N8) | EPI_ISL_14822452 | 2021-03-12 |  | F | 1 | 1 | 1 | 1 | 1 | 1 | 1 | 1 |
|  |  | A/Bar-headed Goose/Tibet/P1987/2021(H5N8) | EPI_ISL_14822547 | 2021-03-12 |  | F | 1 | 1 | 1 | 1 | 1 | 1 | 1 | 1 |
|  |  | A/bar_headed_goose/Tibet/T1640/2021-05-18/H5N8 | EPI_ISL_14822549 | 2021-05-18 | Breeding period in 2021 (May to August) | F | 1 | 1 | 1 | 1 | 1 | 1 | 1 | 1 |
|  |  | A/bar_headed_goose/Tibet/T1707/2021-05-19/H5N8 | EPI_ISL_14822756 | 2021-05-19 |  | F | 1 | 1 | 1 | 1 | 1 | 1 | 1 | 1 |
|  |  | A/brown-headed_gull/Tibet/N38/2021-05-24/H5N8 | EPI_ISL_14822757 | 2021-05-24 |  | L | 1 | - | 1 | - | 1 | 1 | 1 | 1 |
| Central China | Hubei | A/Wild geese/Hubei/H159/2021(H5N8) | EPI_ISL_14823215 | 2021-01-27 | Wintering period in 2020-2021(November in 2020 to February in 2021) | F | 1 | 1 | 1 | 1 | 1 | 1 | 1 | 1 |
|  |  | A/Wild geese/Hubei/H262/2021(H5N8) | EPI_ISL_14823308 | 2021-01-27 |  | F | 1 | 1 | 1 | 1 | 1 | 1 | 1 | 1 |
|  |  | A/Wild geese/Hubei/H358/2021(H5N8) | EPI_ISL_14823405 | 2021-01-27 |  | F | 1 | 1 | 1 | 1 | 1 | 1 | 1 | 1 |
|  |  | A/Wild geese/Hubei/H418/2021(H5N8) | EPI_ISL_14823421 | 2021-01-27 |  | F | 1 | 1 | 1 | 1 | 1 | 1 | 1 | 1 |
|  |  | A/Wild geese/Hubei/H549/2021(H5N8) | EPI_ISL_14823422 | 2021-01-27 |  | F | 1 | 1 | 1 | 1 | 1 | 1 | 1 | 1 |
|  |  | A/Wild geese/Hubei/H68/2021(H5N8) | EPI_ISL_14823423 | 2021-01-27 |  | F | 1 | 1 | 1 | 1 | 1 | 1 | 1 | 1 |
|  | Henan | A/Whooper swan/Sanmenxia/Y1/2020(H5N8) | EPI_ISL_14823424 | 2020-10-30 |  | O | 1 | 1 | 1 | 1 | 1 | 1 | 1 | 1 |
|  |  | A/Whooper swan/Sanmenxia/G2/2020(H5N8) | EPI_ISL_14823425 | 2020-11-04 |  | C | 1 | 1 | 1 | 1 | 1 | 1 | 1 | 1 |
|  |  | A/Whooper swan/Sanmenxia/Y6/2020(H5N8) | EPI_ISL_14823426 | 2020-11-05 |  | O | 1 | 1 | 1 | 1 | 1 | 1 | 1 | 1 |
|  |  | A/Whooper swan/Sanmenxia/Y8/2020(H5N8) | EPI_ISL_14823549 | 2020-11-05 |  | O | 1 | 1 | 1 | 1 | 1 | 1 | 1 | 1 |
|  |  | A/Whooper swan/Sanmenxia/Y10/2020(H5N8) | EPI_ISL_14823646 | 2020-11-08 |  | O | 1 | 1 | 1 | 1 | 1 | 1 | 1 | 1 |
|  |  | A/Whooper swan/Sanmenxia/Y11/2020(H5N8) | EPI_ISL_14823672 | 2020-11-08 |  | O | 1 | 1 | 1 | 1 | 1 | 1 | 1 | 1 |
|  |  | A/Whooper swan/Sanmenxia/Y12/2020(H5N8) | EPI_ISL_14823811 | 2020-11-08 |  | O | 1 | 1 | 1 | 1 | 1 | 1 | 1 | 1 |
|  |  | A/Whooper swan/Sanmenxia/G13/2020(H5N8) | EPI_ISL_14823812 | 2020-11-09 |  | C | 1 | 1 | 1 | 1 | 1 | 1 | 1 | 1 |
|  |  | A/Whooper swan/Sanmenxia/Y14/2020(H5N8) | EPI_ISL_14823813 | 2020-11-09 |  | O | 1 | 1 | 1 | 1 | 1 | 1 | 1 | 1 |
|  |  | A/Whooper swan/Sanmenxia/Y51/2020(H5N8) | EPI_ISL_14823863 | 2020-11-09 |  | O | 1 | - | 1 | 1 | 1 | 1 | 1 | 1 |
|  |  | A/Whooper swan/Sanmenxia/G15/2020(H5N8) | EPI_ISL_14824026 | 2020-11-10 |  | C | 1 | 1 | 1 | 1 | 1 | 1 | 1 | 1 |
|  |  | A/Whooper swan/Sanmenxia/Y15/2020(H5N8) | EPI_ISL_14824091 | 2020-11-10 |  | O | 1 | 1 | 1 | 1 | 1 | 1 | 1 | 1 |
|  |  | A/Whooper swan/Sanmenxia/Y16/2020(H5N8) | EPI_ISL_14824095 | 2020-11-10 |  | O | 1 | 1 | 1 | 1 | 1 | 1 | 1 | 1 |
|  |  | A/Whooper swan/Sanmenxia/Y52/2020(H5N8) | EPI_ISL_14824122 | 2020-11-10 |  | O | 1 | 1 | 1 | 1 | 1 | 1 | 1 | 1 |
|  |  | A/Whooper swan/Sanmenxia/H1/2020(H5N8) | EPI_ISL_14835960 | 2020-11-11 |  | F | 1 | 1 | 1 | 1 | 1 | 1 | 1 | 1 |
|  |  | A/Whooper swan/Sanmenxia/H615/2020(H5N8) | EPI_ISL_14835963 | 2020-11-11 |  | F | 1 | 1 | 1 | 1 | 1 | 1 | 1 | 1 |
|  |  | A/Whooper swan/Sanmenxia/H735/2020(H5N8) | EPI_ISL_14835964 | 2020-11-11 |  | F | 1 | 1 | 1 | 1 | 1 | 1 | 1 | 1 |
|  |  | A/Whooper swan/Sanmenxia/H810/2020(H5N8) | EPI_ISL_14835965 | 2020-11-11 |  | F | 1 | 1 | 1 | 1 | 1 | 1 | 1 | 1 |
|  |  | A/Whooper swan/Sanmenxia/Y24/2020(H5N8) | EPI_ISL_14835968 | 2020-11-12 |  | O | 1 | 1 | 1 | 1 | 1 | 1 | 1 | 1 |
|  |  | A/Whooper swan/Sanmenxia/Y17/2020(H5N8) | EPI_ISL_14835971 | 2020-11-13 |  | O | 1 | - | 1 | - | 1 | - | - | 1 |
|  |  | A/Whooper swan/Sanmenxia/Y18/2020(H5N8) | EPI_ISL_14835974 | 2020-11-13 |  | O | 1 | 1 | 1 | 1 | 1 | 1 | 1 | 1 |
|  |  | A/Whooper swan/Sanmenxia/Y56/2020(H5N8) | EPI_ISL_14835976 | 2020-11-13 |  | O | 1 | 1 | 1 | 1 | 1 | 1 | 1 | 1 |
|  |  | A/Whooper swan/Sanmenxia/Y19/2020(H5N8) | EPI_ISL_14836024 | 2020-11-14 |  | O | 1 | 1 | 1 | 1 | 1 | 1 | 1 | 1 |
|  |  | A/Whooper swan/Sanmenxia/Y20/2020(H5N8) | EPI_ISL_14836025 | 2020-11-14 |  | O | 1 | 1 | 1 | 1 | 1 | 1 | 1 | 1 |
|  |  | A/Whooper swan/Sanmenxia/Y21/2020(H5N8) | EPI_ISL_14836026 | 2020-11-14 |  | O | 1 | 1 | 1 | 1 | 1 | 1 | 1 | 1 |
|  |  | A/Whooper swan/Sanmenxia/Y23-1/2020(H5N8) | EPI_ISL_14836027 | 2020-11-14 |  | O | 1 | 1 | 1 | 1 | 1 | 1 | 1 | 1 |
|  |  | A/Whooper swan/Sanmenxia/Y23-2/2020(H5N8) | EPI_ISL_14836077 | 2020-11-14 |  | O | 1 | 1 | 1 | 1 | 1 | 1 | 1 | 1 |
|  |  | A/Whooper swan/Sanmenxia/G27/2020(H5N8) | EPI_ISL_14836078 | 2020-11-16 |  | C | 1 | 1 | 1 | 1 | 1 | 1 | 1 | 1 |
|  |  | A/Whooper swan/Sanmenxia/G28/2020(H5N8) | EPI_ISL_14836079 | 2020-11-16 |  | C | 1 | 1 | 1 | 1 | 1 | 1 | 1 | 1 |
|  |  | A/Whooper swan/Sanmenxia/G29/2020(H5N8) | EPI_ISL_14836080 | 2020-11-16 |  | C | 1 | 1 | 1 | 1 | 1 | 1 | 1 | 1 |
|  |  | A/Whooper swan/Sanmenxia/Y26/2020(H5N8) | EPI_ISL_14836081 | 2020-11-16 |  | O | 1 | 1 | 1 | 1 | 1 | 1 | 1 | 1 |
|  |  | A/Whooper swan/Sanmenxia/Y27/2020(H5N8) | EPI_ISL_14836082 | 2020-11-16 |  | O | 1 | 1 | 1 | 1 | 1 | 1 | 1 | 1 |
|  |  | A/Whooper swan/Sanmenxia/Y31/2020(H5N8) | EPI_ISL_14836083 | 2020-11-16 |  | O | 1 | 1 | 1 | 1 | 1 | 1 | 1 | 1 |
|  |  | A/Whooper swan/Sanmenxia/Y36/2020(H5N8) | EPI_ISL_14836086 | 2020-11-16 |  | O | 1 | 1 | 1 | 1 | 1 | 1 | 1 | 1 |
|  |  | A/Whooper swan/Sanmenxia/Y48A/2020(H5N8) | EPI_ISL_14836088 | 2020-11-16 |  | O | 1 | 1 | 1 | 1 | 1 | 1 | 1 | 1 |
|  |  | A/Whooper swan/Sanmenxia/Y54/2020(H5N8) | EPI_ISL_14836098 | 2020-11-16 |  | O | 1 | 1 | 1 | 1 | 1 | 1 | 1 | 1 |
|  |  | A/Whooper swan/Sanmenxia/Y57/2020(H5N8) | EPI_ISL_14836101 | 2020-11-16 |  | O | 1 | 1 | 1 | 1 | 1 | 1 | 1 | 1 |
|  |  | A/Whooper swan/Sanmenxia/G48B/2020(H5N8) | EPI_ISL_14836104 | 2020-11-17 |  | C | 1 | 1 | 1 | 1 | 1 | 1 | 1 | 1 |
|  |  | A/Whooper swan/Sanmenxia/Y48B/2020(H5N8) | EPI_ISL_14836369 | 2020-11-17 |  | O | 1 | 1 | 1 | 1 | 1 | 1 | 1 | 1 |
|  |  | A/Whooper swan/Sanmenxia/Y49/2020(H5N8) | EPI_ISL_14836371 | 2020-11-17 |  | O | 1 | 1 | 1 | 1 | 1 | 1 | 1 | 1 |
|  |  | A/Whooper swan/Sanmenxia/G25/2020(H5N8) | EPI_ISL_14836374 | 2020-11-18 |  | C | 1 | 1 | 1 | 1 | 1 | 1 | 1 | 1 |
|  |  | A/Whooper swan/Sanmenxia/Y25/2020(H5N8) | EPI_ISL_14836376 | 2020-11-18 |  | O | 1 | 1 | 1 | 1 | 1 | 1 | 1 | 1 |
|  |  | A/Whooper swan/Sanmenxia/B560/2020(H5N8) | EPI_ISL_14836426 | 2020-12-01 |  | O | 1 | 1 | 1 | 1 | 1 | 1 | 1 | 1 |
|  |  | A/Whooper swan/Sanmenxia/B312/2020(H5N8) | EPI_ISL_14836593 | 2020-12-06 |  | O | 1 | 1 | 1 | 1 | 1 | 1 | 1 | 1 |
|  |  | A/Whooper swan/Sanmenxia/B497/2020(H5N8) | EPI_ISL_14838179 | 2020-12-12 |  | O | 1 | 1 | 1 | 1 | 1 | 1 | 1 | 1 |
|  |  | A/Whooper swan/Sanmenxia/B1109/2021(H5N8) | EPI_ISL_14839943 | 2021-01-15 |  | O | 1 | 1 | 1 | 1 | 1 | 1 | 1 | 1 |
|  |  | A/Whooper swan/Sanmenxia/B1770/2020(H5N8) | EPI_ISL_14840034 | 2021-01-15 |  | F | 1 | 1 | 1 | 1 | 1 | 1 | 1 | 1 |
|  |  | A/Whooper swan/Sanmenxia/B2465/2021(H5N8) | EPI_ISL_14840053 | 2021-01-21 |  | O | 1 | 1 | 1 | 1 | 1 | 1 | 1 | 1 |
|  |  | A/Whooper swan/Sanmenxia/B413/2021(H5N8) | EPI_ISL_14840107 | 2021-03-15 |  | O | 1 | 1 | 1 | 1 | 1 | 1 | 1 | 1 |
| Western China | Qinghai | A/Bar-headed Goose/Qinghai/Q11/2020(H5) | EPI_ISL_14845547 | 2020-09-24 | Autumn migration period in 2020 (September and October) | F | 1 | 1 | - | - | 1 | - | - | 1 |
| Central China | Henan | A/Whooper swan/Sanmenxia/Y41/2020(H5) | EPI_ISL_14845578 | 2020-11-06 | Wintering period in 2020-2021(November in 2020 to February in 2021) | O | - | - | - | - | 1 | - | - | - |
| Eastern China | Shandong | A/whooper_swan/Rongcheng/M82/2021-04-29/H5 | EPI_ISL_14845579 | 2021-04-29 | Spring migration period in 2021(March and April) | L | 1 | - | 1 | 1 | 1 | - | - | 1 |
| Northern China | Inner Mongolia | A/wild bird/Tumuji/TJ318/2020(H5) | EPI_ISL_14846092 | 2020-04-25 | - | F | 1 | - | - | - | 1 | - | - | - |

*: F, faecal; O, oropharyngeal swab; C, cloacal swab; L, lungs of dead wild birds.

⁑: 1, represent the segment was obtained; 0, represent the segment was missing.

Table S2. Annual migration patterns of waterfowl birds between regions in Eurasian.

| **Season** | **Migration route** | **Species (number of migration route)** | **Total number of migration route** | **Year** | **Reference** |
| --- | --- | --- | --- | --- | --- |
| Spring migration period | **China** → **North Asia** (Mongolia, Kazakhstan, Russia) | Whooper swan *Cygnus cygnus* (169), Tundra swan *Cygnus columbianus* (1), Common teal *Anas crecca* (5), Bar-headed goose *Anser indicus* (1), Common shelduck *Tadorna tadorna* (2), Mallard *Anas platyrhynchos* (38), | 216 | 2015-2022 | GPS |
|  | **South Asia** (India, Sri Lanka, Myanmar, Thailand, Vietnam, Indonesia, Philippines) → **China** → **North Asia** (Mongolia) | Black-winged stilt *Himantopus himantopus* (1), Black-tailed godwit *Limosa limosa* (5) | 6 | 2020-2021 | GPS |
|  | **East Asia** (North Korea, South Korea, Japan) → **China** | Hooded crane *Grus monachal* (6) | 6 | 2012-2014 | GPS |
|  | **East Asia** (North Korea, South Korea) → **China** → **North Asia** (Mongolia) | Common shelduck *Tadorna tadorna* (2) | 2 | 2019-2021 | GPS |
|  | **South Asia** (India, Bangladesh) → **China** | Great black-headed gull *Larus ichthyaetus* (4) | 4 | 2007 | Chu et al. (*1*) |
|  | **China** → **North Asia** (Kazakhstan, Mongolia, Russia) | Tundra swan *Cygnus columbianus* (1) | 1 | 2015 | Huang et al. (*2*) |
|  | **East Asia** (Japan) → **North Asia** (Russia) | Whooper swan *Cygnus cygnus* (57), Tundra swan *Cygnus columbianus* (17) | 74 | 2009-2012 | Shimada et al. (*3*), Chen et al. (*4*) |
|  | **South Asia** (India) → **North Asia** (Kazakhstan) | Sociable lapwing *Vanellus gregarious* (3) | 3 | 2007-2015 | Donald et al. (*5*) |
|  | **South Asia** (Iran) → **Europe** (Armenia) | White stork *Ciconia ciconia* (2) | 2 | 2014 | Flack et al. (*6*) |
|  | **Europe** (Italy, Netherlands) → **North Asia** (Russia) | Barnacle goose *Branta leucopsis* (12), Eurasian teals *Anas crecca* (17) | 29 | 2008-2016 | Kölzsch et al. (*7*), Giunchi et al. (*8*) |
|  | **Africa** (Egypt, Sudan) → **Europe** (Turkey, Armenia) → **North Asia** (Kazakhstan, Russia) | Common shelduck *Tadorna tadorna* (1), Northern shoveler *Spatula clypeata* (1), Sociable lapwing *Vanellus gregarious* (5) | 7 | 2005-2015 | Iverson et al. (*9*), Donald et al. (*5*) |
|  | **Africa** (South Africa) → **South Asia** (Syria) → **Europe** (Turkey, Bulgaria, Romania, Ukraine) → **North Asia** (Russia) | White stork *Ciconia ciconia* (4) | 4 | 2014 | Flack et al. (*6*) |
|  | **Africa** (South Africa) → **South Asia** (Syria) → **Europe** (Poland) | White stork *Ciconia ciconia* (2) | 2 | 2014 | Flack et al. (*6*) |
| Autumn migration period | **North Asia** (Russia, Kazakhstan, Mongolia) → **China** | Whooper swan *Cygnus cygnus* (65), Black-headed gull *Chroicocephalus ridibundus* (2), Bar-headed goose *Anser indicus* (1), Common shelduck *Tadorna tadorna* (4), Common pochard *Aythya ferina* (1), Common teal *Anas crecca* (6), Common redshank *Tringa tetanus* (1), Mallard *Anas platyrhynchos* (20) | 100 | 2015-2021 | GPS |
|  | **North Asia** (Mongolia) → **China** → **South Asia** (India, Sri Lanka, Myanmar, Thailand, Vietnam, Indonesia, the Philippines) | Asian dowitcher *Limnodromus semipalmatus* (5), Black-tailed godwit *Limosa limosa* (16), Bar-headed goose *Anser indicus* (3) | 24 | 2006-2021 | GPS |
|  | **North Asia** (Mongolia) → **China** → **East Asia** (North Korea, South Korea) | Common shelduck *Tadorna tadorna* (2) | 2 | 2019-2021 | GPS |
|  | **China** → **East Asia** (North Korea, South Korea, Japan) | Hooded crane *Grus monachal* (8) | 8 | 2011-2014 | GPS |
|  | **China** → **East Asia** (North Korea) → **China** | Black-faced spoonbill *Platalea minor* (1) | 1 | 2018 | GPS |
|  | **China** → **South Asia** (Uzbekistan, India) | Bar-headed goose *Anser indicus* (3) | 3 | 2006 | GPS |
|  | **China** → **South Asia** (India, Bangladesh) | Great black-headed gull *Larus ichthyaetus* (4) | 4 | 2007 | Chu et al. (*1*) |
|  | **North Asia** (Kazakhstan, Mongolia, Russia) → **China** | Tundra swan *Cygnus columbianus* (1) | 1 | 2015 | Huang et al. (*2*) |
|  | **North Asia** (Russia) → **East Asia** (Japan) | Whooper swan *Cygnus cygnus* (33), Tundra swan *Cygnus columbianus* (6) | 39 | 2009-2012 | Shimada et al. (*3*), Chen et al. (*4*) |
|  | **North Asia** (Kazakhstan) → **South Asia** (India) | Sociable lapwing *Vanellus gregarious* (5) | 5 | 2007-2015 | Donald et al. (*5*) |
|  | **East Asia** (North Korea, South Korea) → **China** → **South Asia** (Vietnam, Cambodia) | Black-faced spoonbill *Platalea minor* (1) | 1 | 2010 | Wood et al. (*10*) |
|  | **Europe** (Armenia) → **South Asia** (Iran) | White stork *Ciconia ciconia* (2) | 2 | 2013 | Flack et al. (*6*) |
|  | **Europe** (Poland) → **South Asia** (Syria) →**Africa** (South Africa) | White stork *Ciconia ciconia* (2) | 2 | 2013 | Flack et al. (*6*) |
|  | **North Asia** (Kazakhstan, Russia) → **Europe** (Turkey, Armenia) → **Africa** (Sudan) | Sociable lapwing *Vanellus gregarious* (11) | 11 | 2007-2015 | Donald et al. (*5*) |
|  | **North Asia** (Russia) → **Europe** (Turkey, Bulgaria, Romania, Ukraine) → **South Asia** (Syria) →**Africa** (South Africa) | White stork *Ciconia ciconia* (7) | 7 | 2013 | Flack et al. (*6*) |

Table S3. Species and number of individuals of the bird trajectory dataset in China.

| **Type** | **Species** | **Number** | **Capture location** | **Capture year** |
| --- | --- | --- | --- | --- |
| Cranes | Hooded crane  *Grus monacha* | 4 | Xinqing, Heilongjiang of eastern China | 2011 |
| Storks | Black-faced spoonbill  *Platalea minor* | 10 | Zhuanghe, Liaoning of eastern China | 2017-2018 |
| Geese and ducks | Whooper swan  *Cygnus cygnus* | 3 | Manas, Xinjiang of western China | 2013 |
|  |  | 10 | Manas, Xinjiang of western China | 2021 |
|  |  | 13 | Manas, Xinjiang of western China | 2022 |
|  |  | 9 | Bayanbulak, Xinjiang of western China | 2018 |
|  |  | 239 | Sanmenxia Reservoir Area, Henan of central China | 2015-2021 |
|  |  | 5 | Rongcheng, Shandong of eastern China | 2016 |
|  | Tundra swan  *Cygnus columbianus* | 9 | Longgan Lake, Hubei of central China | 2018 |
|  |  | 10 | Longgan Lake, Hubei of central China | 2021 |
|  | Mallard  *Anas platyrhynchos* | 34 | Tianhu Lake, Ningxia of western China | 2019 |
|  |  | 2 | Wang Lake, Hubei of central China | 2018 |
|  | Bean goose  *Anser fabalis* | 4 | Longgan Lake, Hubei of central China | 2018 |
|  | Spot-billed duck  *Anas zonorhyncha* | 6 | Tianhu Lake, Ningxia of western China | 2019 |
|  | Bar-headed goose  *Anser indicus* | 6 | Qinghai Lake, Qinghai of western China | 2006 |
|  |  | 4 | Qinghai Lake, Qinghai of western China | 2007 |
|  |  | 16 | Caohai Nature Reserve, Guizhou of western China | 2019 |
|  | Common shelduck  *Tadorna tadorna* | 4 | Ulz River, Dornod Aymag of eastern Mongolia | 2019 |
|  | Common pochard  *Aythya ferina* | 5 | Tianhu Lake, Ningxia of western China | 2019 |
|  |  | 4 | Ulz River, Dornod Aymag of eastern Mongolia | 2019 |
|  | Common teal  *Anas crecca* | 6 | Wang Lake, Hubei of central China | 2021 |
|  |  | 4 | Ulz River, Dornod Aymag of eastern Mongolia | 2019 |
|  | Gadwall  *Anas strepera* | 10 | Tianhu Lake, Ningxia of western China | 2019 |
|  | Northern shoveler  *Spatula clypeata* | 9 | Tianhu Lake, Ningxia of western China | 2019 |
|  | Ferruginous duck  *Aythya nyroca* | 7 | Tianhu Lake, Ningxia of western China | 2019 |
| Gulls | Brown-headed gull  *Chroicocephalus brunnicephalus* | 10 | Qinghai Lake, Qinghai of western China | 2021 |
|  | Black-headed gull  *Chroicocephalus ridibundus* | 22 | Dianchi Lake, Yunnan of western China | 2018 |
|  |  | 69 | Dianchi Lake, Yunnan of western China | 2019 |
|  |  | 4 | Ulz River, Dornod Aymag of eastern Mongolia | 2019 |
| Shorebirds | Common redshank  *Tringa totanus* | 2 | Ulz River, Dornod Aymag of eastern Mongolia | 2019 |
|  | Black-winged stilt  *Himantopus himantopus* | 4 | Ulz River, Dornod Aymag of eastern Mongolia | 2019 |
|  | Asian dowitcher  *Limnodromus semipalmatus* | 6 | Ulz River, Dornod Aymag of eastern Mongolia | 2019 |
|  | Black-tailed godwit  *Limosa limosa* | 20 | Ulz River, Dornod Aymag of eastern Mongolia | 2019 |

Table S4. Geographic information for genetic datasets.

| **Region^a^** | **Dataset1^b^** | **Dataset2^c^** | **Dataset3^d^** | **Dataset4^e^** |
| --- | --- | --- | --- | --- |
| China | China | China | China | China |
| East Asia | Japan | Japan | Japan | Japan |
|  | South Korea | South Korea | South Korea | South Korea |
| North Asia | Kazakhstan | Kazakhstan | Kazakhstan | Kazakhstan |
|  | Russia | Russia | Russia | Russia |
| South Asia | India |  | India | India |
|  | Iran |  | Iran | Iran |
|  | Iraq | Iraq | Iraq | Iraq |
|  | Israel |  |  |  |
|  | Pakistan |  | Pakistan | Pakistan |
|  | Saudi Arabia |  | Saudi Arabia |  |
| Europe | Albania | Albania | Albania | Albania |
|  | Austria | Austria | Austria | Austria |
|  | Belgium | Belgium | Belgium | Belgium |
|  | Bulgaria | Bulgaria | Bulgaria | Bulgaria |
|  | Croatia | Croatia | Croatia | Croatia |
|  | Czech Republic | Czech Republic | Czech Republic | Czech Republic |
|  | Denmark | Denmark | Denmark | Denmark |
|  | France |  |  |  |
|  | Germany | Germany | Germany | Germany |
|  | Greece |  | Greece | Greece |
|  | Hungary | Hungary | Hungary | Hungary |
|  | Ireland | Ireland |  |  |
|  | Italy | Italy | Italy | Italy |
|  | Latvia | Latvia |  | Latvia |
|  |  |  | Luxembourg |  |
|  | Lithuania |  |  |  |
|  | Netherlands | Netherlands | Netherlands | Netherlands |
|  | Norway | Norway | Norway | Norway |
|  | Poland | Poland | Poland | Poland |
|  | Romania | Romania | Romania | Romania |
|  | Serbia | Serbia | Serbia | Serbia |
|  | Slovenia | Slovakia | Slovakia | Slovakia |
|  |  | Slovenia |  | Slovenia |
|  |  | Spain |  | Spain |
|  | Sweden |  | Sweden | Sweden |
|  | Switzerland |  |  |  |
|  |  |  | Ukraine |  |
|  | United Kingdom | United Kingdom | United Kingdom | United Kingdom |

^a^All gene sequences from different countries were divided into five groups: 1 Europe-group (Europe), 4 Asia-groups (including Asia-group China, Asia-group East Asia, Asia-group South Asia, Asia-group North Asia).

^b^501 HA segment gene sequences of HPAI H5N8 virus for the sub-sampled dataset.

^c^249 HA segment gene sequences of HPAI H5N8 virus for the uneven-sampled dataset.

^d^484 NA segment gene sequences of HPAI H5N8 virus for the sub-sampled dataset.

^e^279 NA segment gene sequences of HPAI H5N8 virus for the uneven-sampled dataset.

Table S5. Inferred Bayes factor values from the joint analysis of the uneven-sampled of HA and NA gene datasets by a Bayesian stochastic search variable selection procedure.

| **Origin** | **Destination** | **Bayes factor** | **Posterior probability** |
| --- | --- | --- | --- |
| China | East Asia | 50703.78677 | 1 |
| North Asia | Europe | 50703.78677 | 1 |
| North Asia | China | 870.9979209 | 0.996269377 |
| Europe | North Asia | 158.2258823 | 0.979803177 |
| East Asia | Europe | 82.68261701 | 0.962050556 |
| North Asia | South Asia | 49.55830932 | 0.938251753 |
| China | North Asia | 18.87161806 | 0.852640381 |
| East Asia | China | 3.656209262 | 0.528526404 |
| China | Europe | 3.46621688 | 0.515211938 |
| Europe | China | 3.443974915 | 0.513603911 |
| Europe | East Asia | 2.414205899 | 0.425355374 |
| South Asia | Europe | 1.974094421 | 0.377050235 |
| South Asia | North Asia | 1.780934353 | 0.35318711 |
| Europe | South Asia | 1.459354434 | 0.309127163 |
| South Asia | East Asia | 1.145476577 | 0.259921528 |
| South Asia | China | 0.946521139 | 0.224930855 |
| East Asia | North Asia | 0.409432972 | 0.111532772 |
| North Asia | East Asia | 0.288386709 | 0.081237538 |
| East Asia | South Asia | 0.266896 | 0.075641603 |
| China | South Asia | 0.230688295 | 0.06605776 |

Table S6. Inferred Bayes factor values under the uneven-sampled HA gene dataset by a Bayesian stochastic search variable selection procedure.

| **Origin** | **Destination** | **Bayes factor** | **Posterior probability** |
| --- | --- | --- | --- |
| China | East Asia | 39940.72899 | 1 |
| North Asia | Europe | 39940.72899 | 1 |
| North Asia | China | 884.3827011 | 0.996325631 |
| Europe | North Asia | 109.5745987 | 0.971094962 |
| China | Europe | 19.77421735 | 0.858414306 |
| Europe | South Asia | 8.935105664 | 0.732587572 |
| East Asia | Europe | 8.438523142 | 0.721237854 |
| North Asia | South Asia | 3.482345527 | 0.516371356 |
| South Asia | North Asia | 2.698477859 | 0.452763942 |
| East Asia | China | 2.658726789 | 0.449089573 |
| South Asia | China | 1.306104904 | 0.285947579 |
| South Asia | Europe | 1.247836328 | 0.27672083 |
| Europe | East Asia | 1.243767418 | 0.276067608 |
| South Asia | East Asia | 1.231604629 | 0.274107945 |
| Europe | China | 1.032135027 | 0.240385401 |
| East Asia | North Asia | 0.766703574 | 0.190332326 |
| China | North Asia | 0.448665231 | 0.120927574 |
| North Asia | East Asia | 0.430489753 | 0.116599984 |
| East Asia | South Asia | 0.424697544 | 0.115211889 |
| China | South Asia | 0.297269059 | 0.083530661 |

Table S7. Inferred Bayes factor values under the sub-sampled HA gene dataset by a Bayesian stochastic search variable selection procedure.

| **Origin** | **Destination** | **Bayes factor** | **Posterior probability** |
| --- | --- | --- | --- |
| China | East Asia | 15694.49517 | 0.999792229 |
| North Asia | South Asia | 15694.49517 | 1 |
| North Asia | China | 15694.49517 | 0.999792229 |
| North Asia | Europe | 15694.49517 | 1 |
| Europe | North Asia | 487.2933644 | 0.99335134 |
| Europe | East Asia | 26.926461 | 0.891959277 |
| China | North Asia | 22.98889661 | 0.875753169 |
| South Asia | China | 18.78587844 | 0.852067318 |
| East Asia | Europe | 9.187124518 | 0.738001247 |
| East Asia | China | 7.571977831 | 0.69894037 |
| East Asia | North Asia | 1.695131911 | 0.341990443 |
| South Asia | East Asia | 1.384148517 | 0.297943071 |
| North Asia | East Asia | 1.082063548 | 0.249116975 |
| South Asia | Europe | 0.892411954 | 0.214834822 |
| China | South Asia | 0.840311263 | 0.204861833 |
| Europe | China | 0.666834881 | 0.169748598 |
| South Asia | North Asia | 0.610632287 | 0.157697902 |
| China | Europe | 0.423386752 | 0.114897154 |
| Europe | South Asia | 0.331453537 | 0.092250156 |
| East Asia | South Asia | 0.330631343 | 0.092042385 |

Table S8. Inferred Bayes factor values under the sub-sampled NA gene dataset by a Bayesian stochastic search variable selection procedure.

| **Origin** | **Destination** | **Bayes factor** | **Posterior probability** |
| --- | --- | --- | --- |
| North Asia | Europe | 56887.6527 | 1 |
| North Asia | South Asia | 4737.647987 | 0.999312045 |
| China | East Asia | 3157.344814 | 0.998968067 |
| North Asia | China | 1419.011323 | 0.997706816 |
| China | North Asia | 101.7032537 | 0.968927363 |
| East Asia | Europe | 55.26821449 | 0.944275641 |
| Europe | North Asia | 43.37036425 | 0.930057903 |
| South Asia | China | 12.95594574 | 0.798887806 |
| South Asia | East Asia | 10.06186646 | 0.75520266 |
| East Asia | China | 3.706123704 | 0.531903916 |
| Europe | China | 2.340172165 | 0.417760706 |
| China | South Asia | 0.898376246 | 0.215960557 |
| North Asia | East Asia | 0.843743296 | 0.205526572 |
| East Asia | North Asia | 0.806462635 | 0.198245715 |
| South Asia | Europe | 0.721309275 | 0.181104168 |
| South Asia | North Asia | 0.552553837 | 0.144871868 |
| China | Europe | 0.548211812 | 0.143897265 |
| Europe | East Asia | 0.544898104 | 0.143151981 |
| East Asia | South Asia | 0.435803675 | 0.117869633 |
| Europe | South Asia | 0.388362393 | 0.106403715 |

Table S9. Inferred Bayes factor values under the uneven-sampled NA gene dataset by a Bayesian stochastic search variable selection procedure.

| **Origin** | **Destination** | **Bayes factor** | **Posterior probability** |
| --- | --- | --- | --- |
| North Asia | Europe | 54095.78074 | 1 |
| China | East Asia | 440.1732401 | 0.992644842 |
| China | North Asia | 37.72259027 | 0.920419606 |
| North Asia | South Asia | 31.61857196 | 0.906493037 |
| East Asia | Europe | 21.70341532 | 0.869355519 |
| Europe | China | 19.32679397 | 0.855609815 |
| North Asia | China | 5.216612185 | 0.615301139 |
| Europe | North Asia | 5.196729106 | 0.614396817 |
| South Asia | Europe | 2.288794851 | 0.412371134 |
| South Asia | North Asia | 2.156498612 | 0.398022548 |
| East Asia | China | 1.155076226 | 0.261530114 |
| South Asia | East Asia | 1.054650701 | 0.244347983 |
| South Asia | China | 0.928294032 | 0.221559052 |
| East Asia | North Asia | 0.847775524 | 0.206306143 |
| Europe | South Asia | 0.689032715 | 0.174413697 |
| Europe | East Asia | 0.585920739 | 0.152287936 |
| China | South Asia | 0.520030477 | 0.137517333 |
| North Asia | East Asia | 0.406199017 | 0.110749382 |
| East Asia | South Asia | 0.400736649 | 0.109423042 |
| China | Europe | 0.379784042 | 0.104298547 |

**References**

1. G. Chu *et al.*, Satellite-Tracking Migration Route of Great Black-Headed Gulls Breeding at Qinghai Lake, China. *Scientia Silvae Sinicae* **44**, 99-104 (2008).

2. X. Z. Huang Tian, Peng Jiao, Zhao Yunlin, Study on the Migration Routes of Overwintering Cygnus Columbianus in Dongting Lake Based on Satellite Tracking. *Sichuan Journal of Zoology* **37**, 361-372 (2018).

3. T. Shimada *et al.*, Satellite tracking of migrating Whooper Swans Cygnus cygnus wintering in Japan. *Ornithological Science* **13**, 67-75 (2014).

4. W. B. Chen *et al.*, Migration of Tundra Swans (Cygnus columbianus) Wintering in Japan Using Satellite Tracking: Identification of the Eastern Palearctic Flyway. *Zoological Science* **33**, 63-72 (2016).

5. P. F. Donald *et al.*, Migration strategy, site fidelity and population size of the globally threatened Sociable Lapwing Vanellus gregarius. *Journal of Ornithology* **162**, 349-367 (2021).

6. A. Flack *et al.*, Costs of migratory decisions: A comparison across eight white stork populations. *Science Advances* **2**, (2016).

7. A. Kolzsch *et al.*, Forecasting spring from afar? Timing of migration and predictability of phenology along different migration routes of an avian herbivore. *Journal of Animal Ecology* **84**, 272-283 (2015).

8. D. Giunchi *et al.*, Spring migratory routes and stopover duration of satellite-tracked Eurasian Teals Anas crecca wintering in Italy. *Ibis* **161**, 117-130 (2019).

9. S. A. Iverson *et al.*, Migratory movements of waterfowl in Central Asia and avian influenza emergence: sporadic transmission of H5N1 from east to west. *Ibis* **153**, 279-292 (2011).

10. C. Wood *et al.*, New perspectives on habitat selection by the Black-faced Spoonbill Platalea minor based upon satellite telemetry. *Bird Conservation International* **23**, 495-501 (2013).
